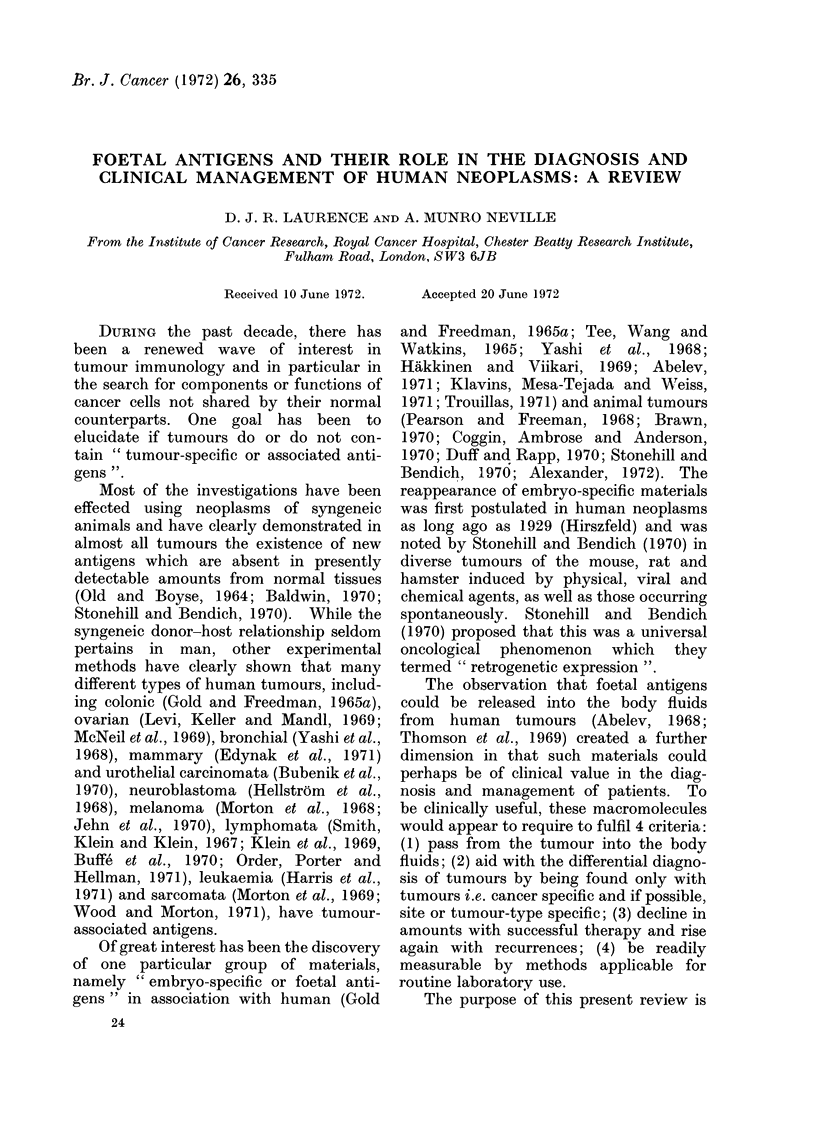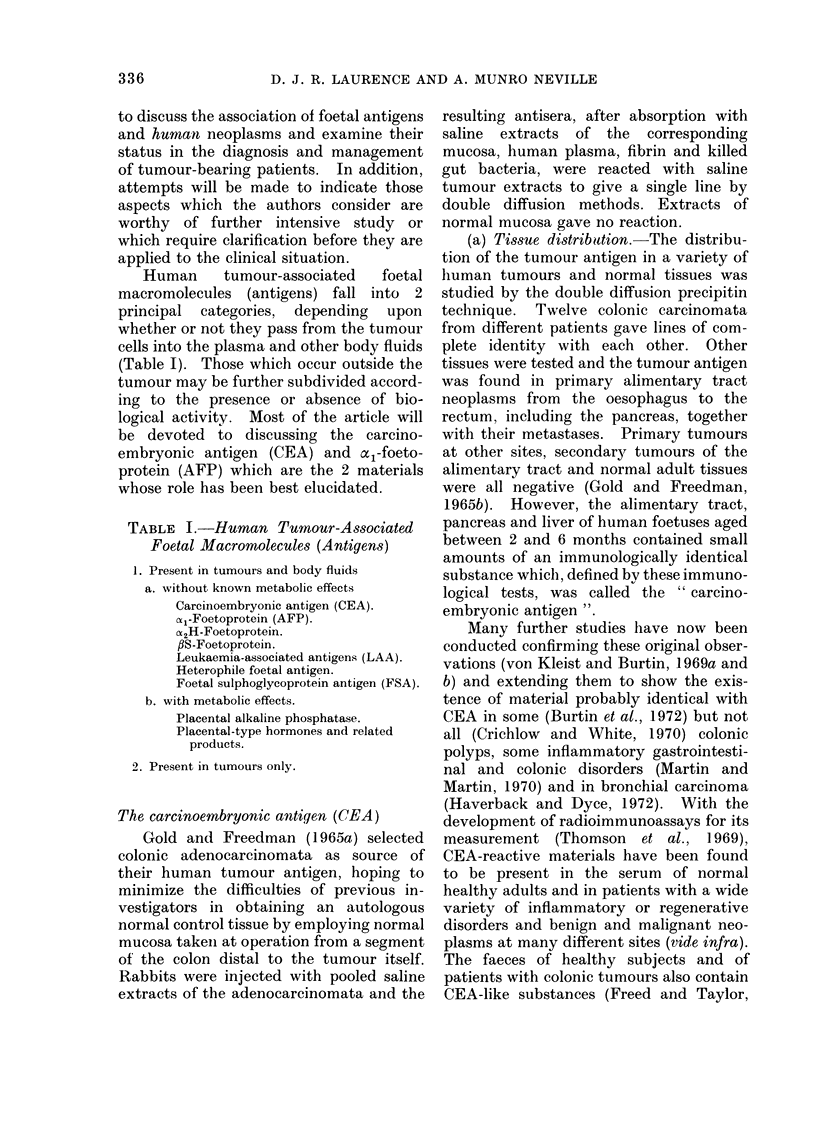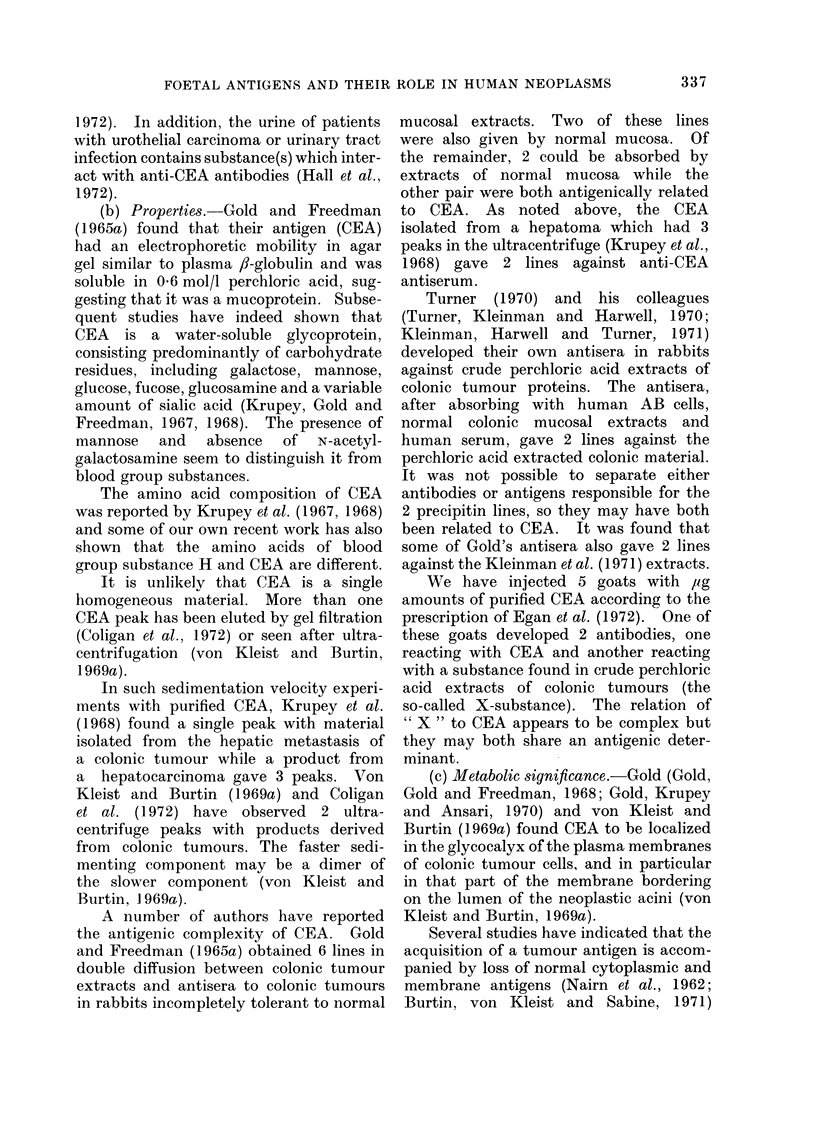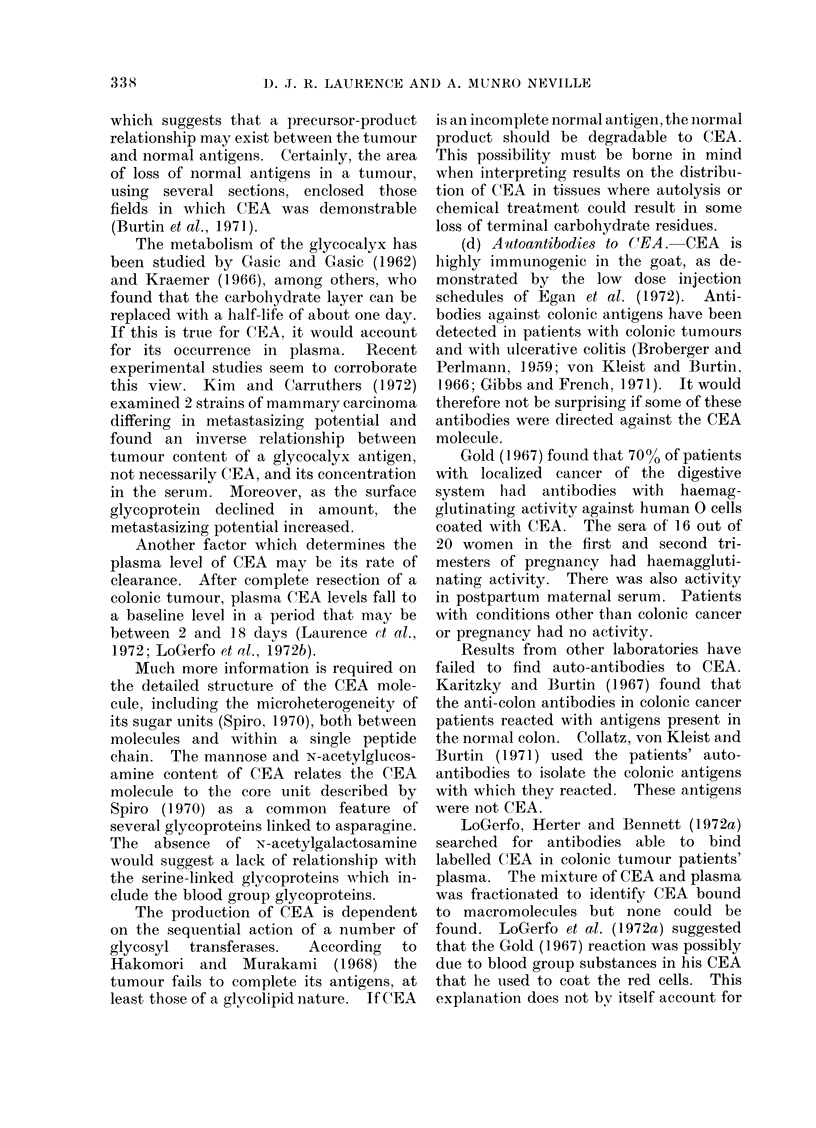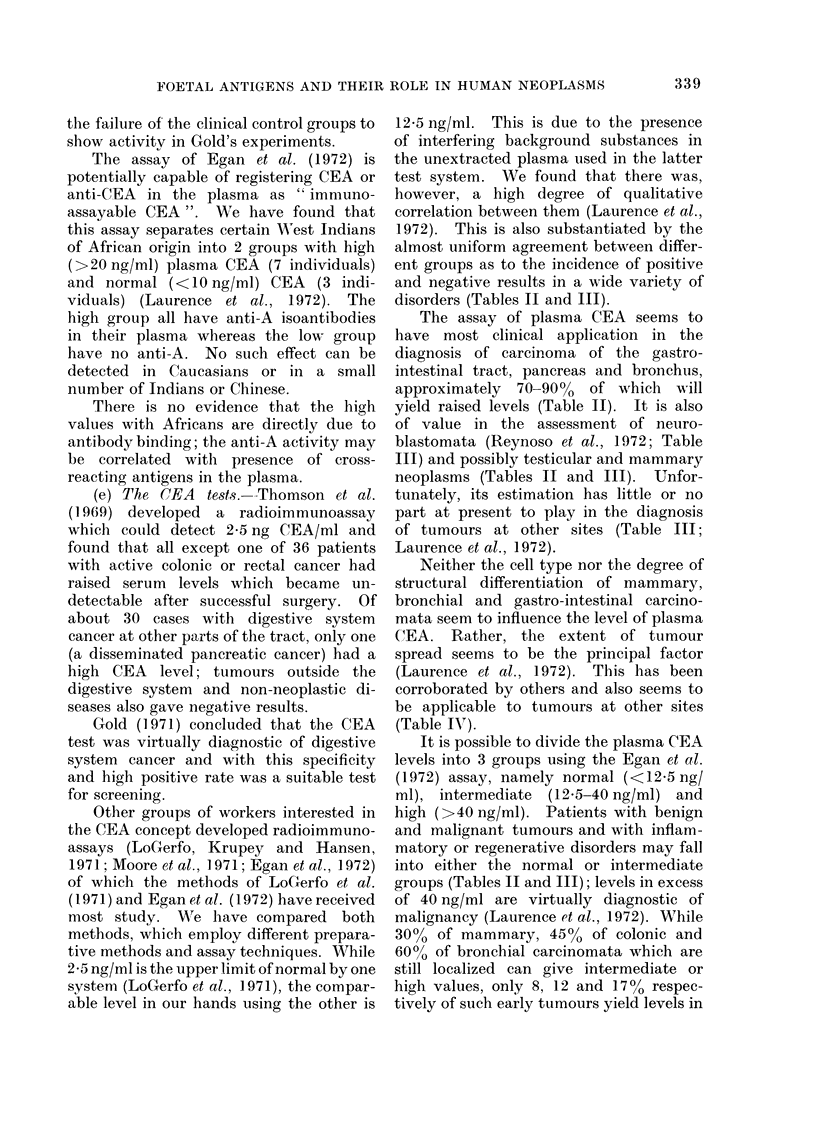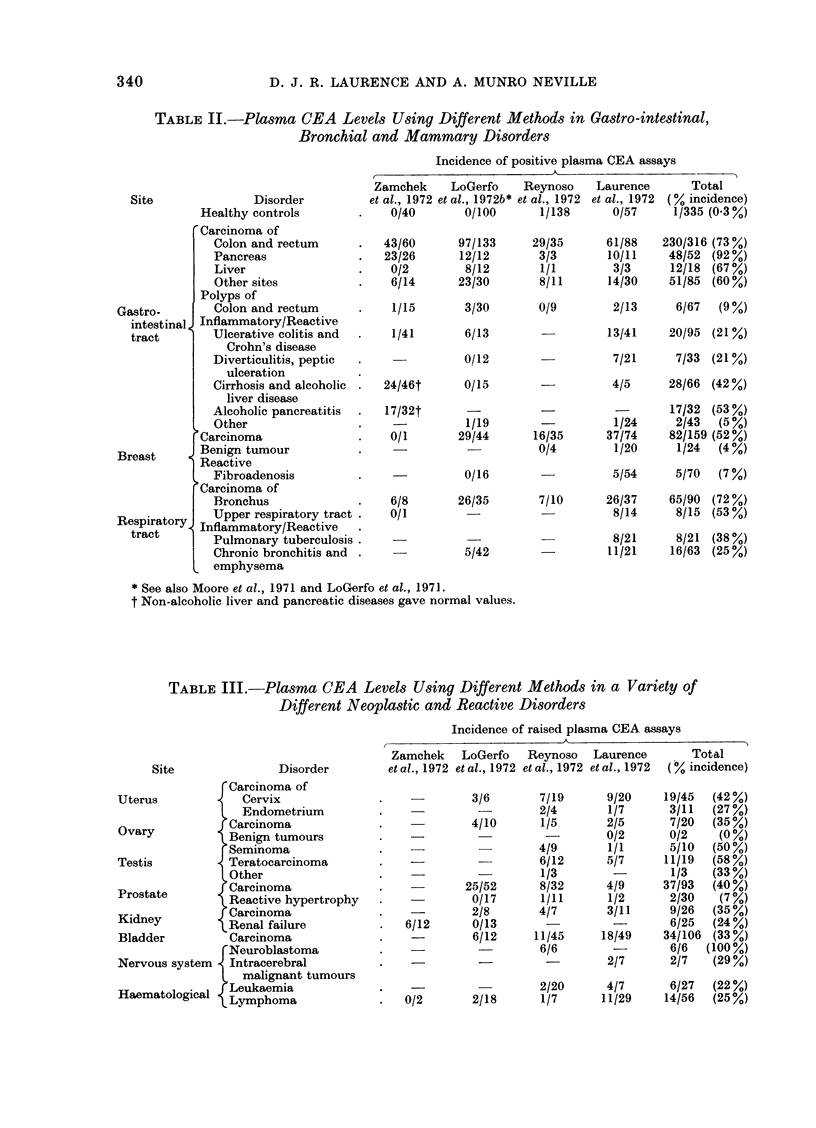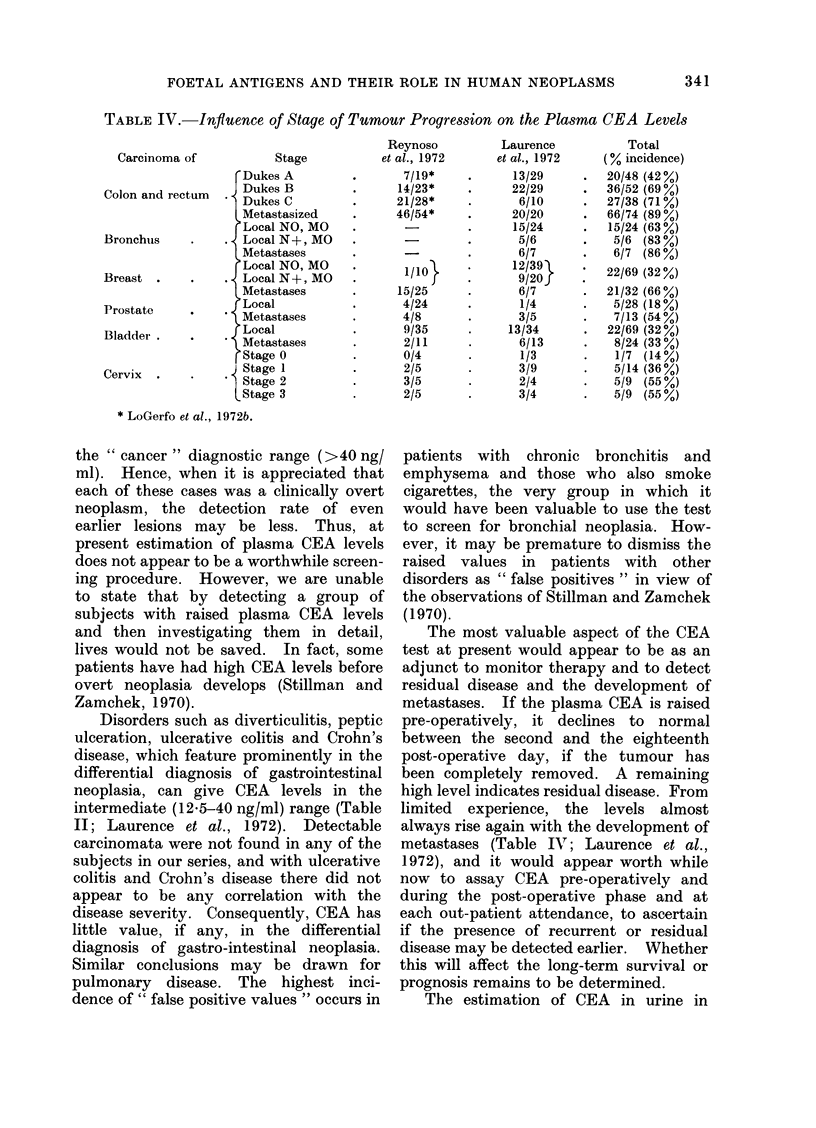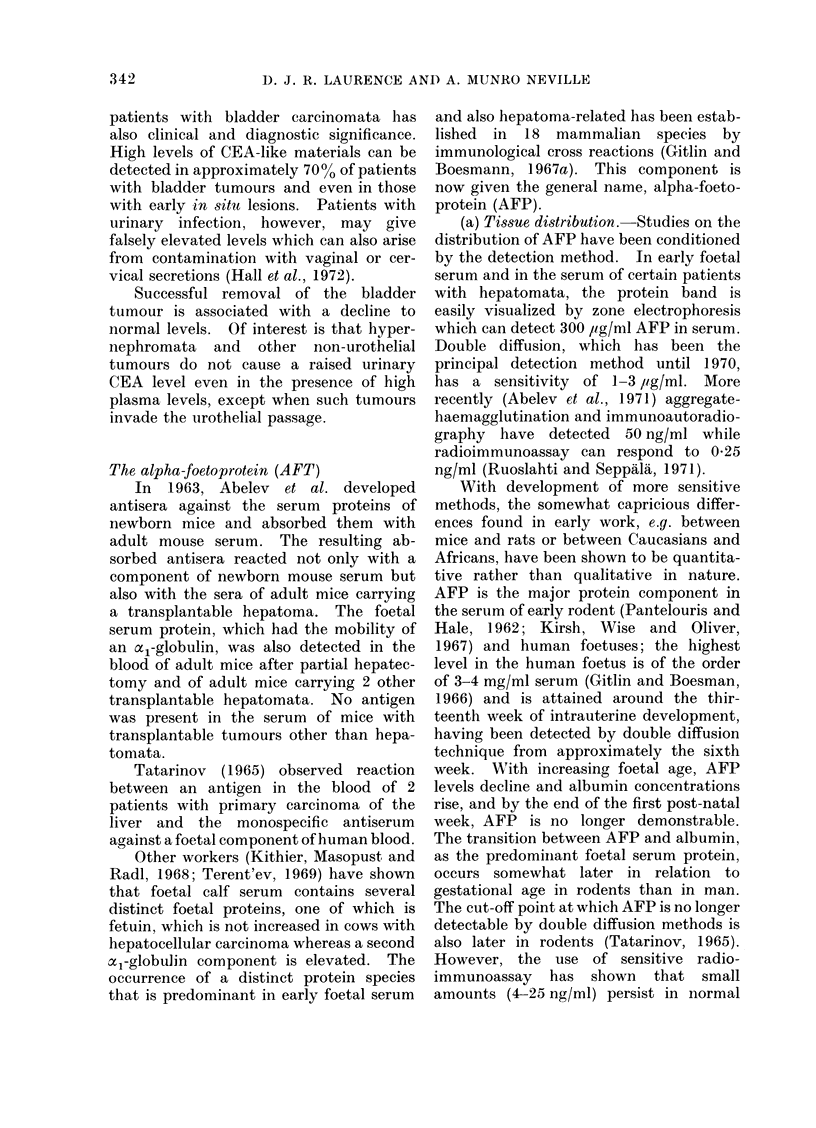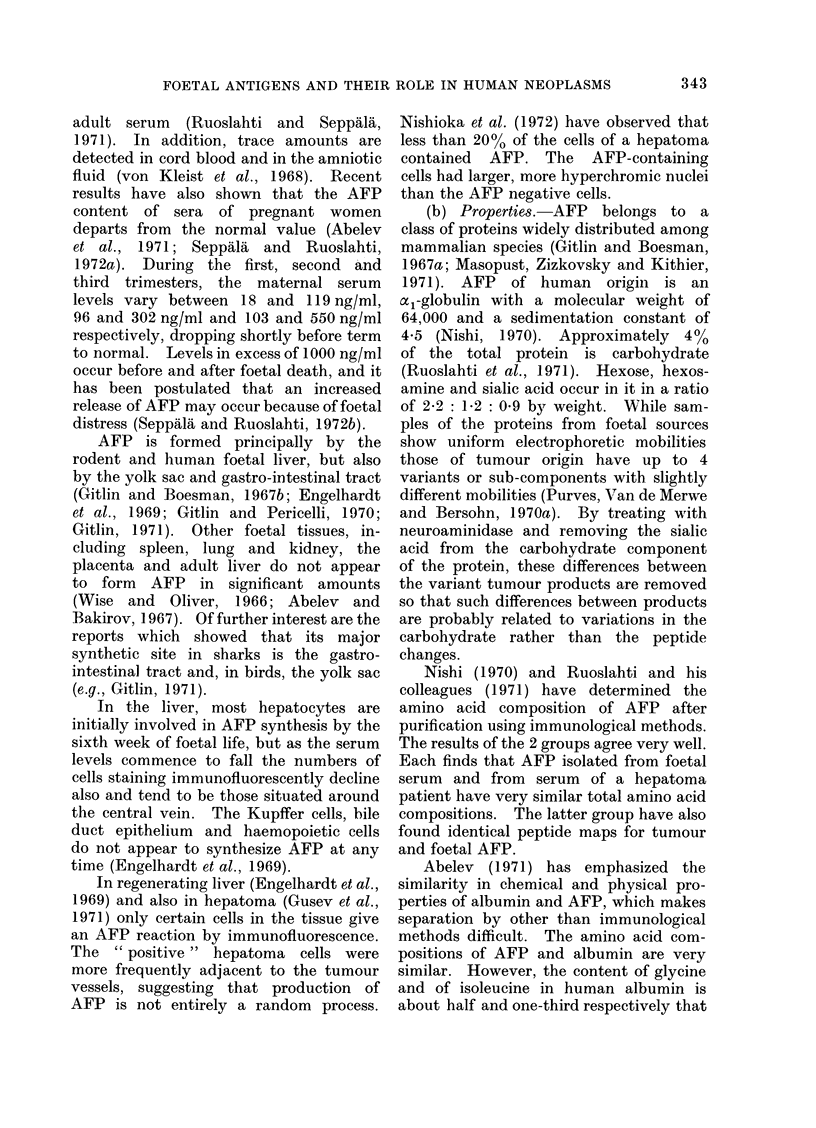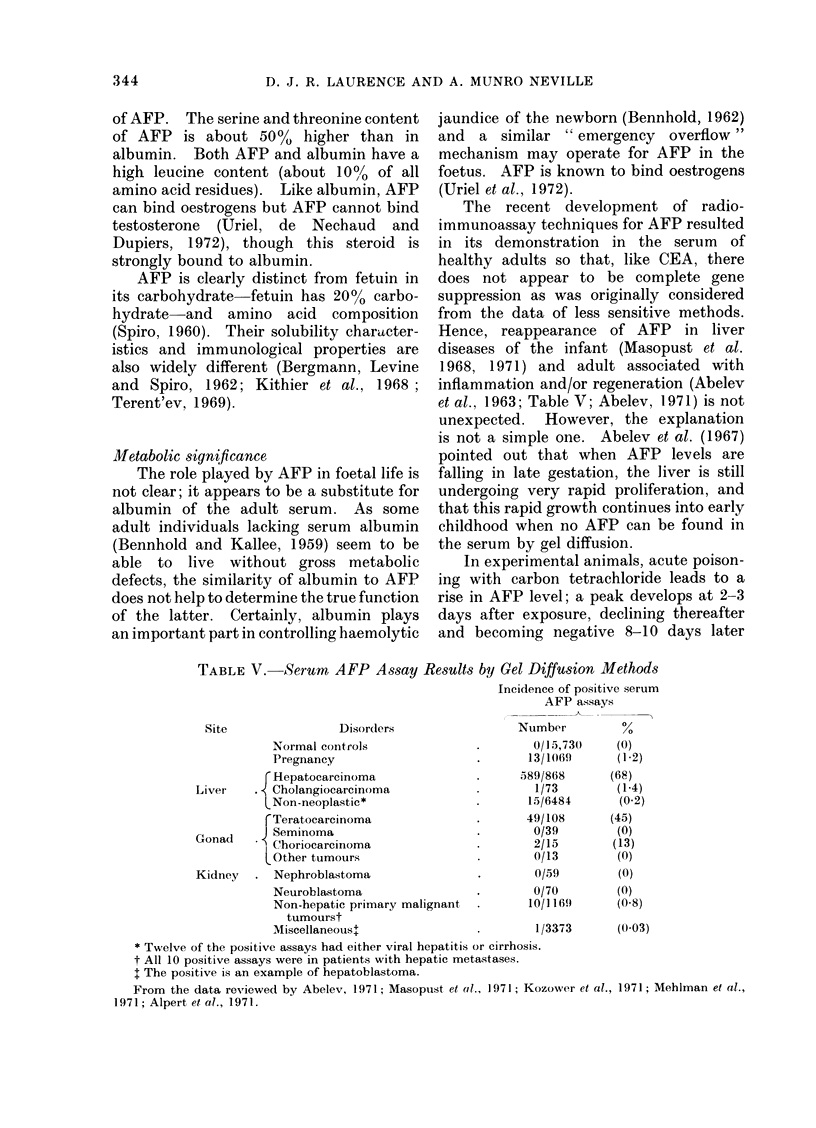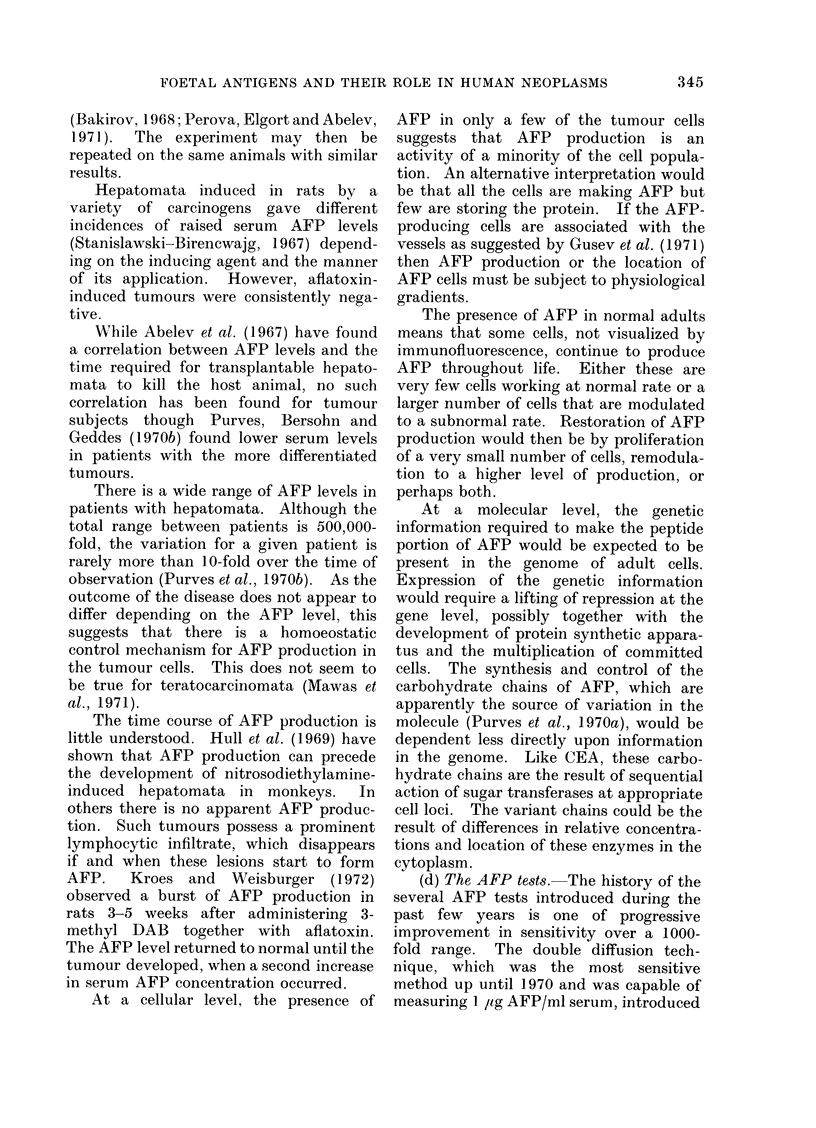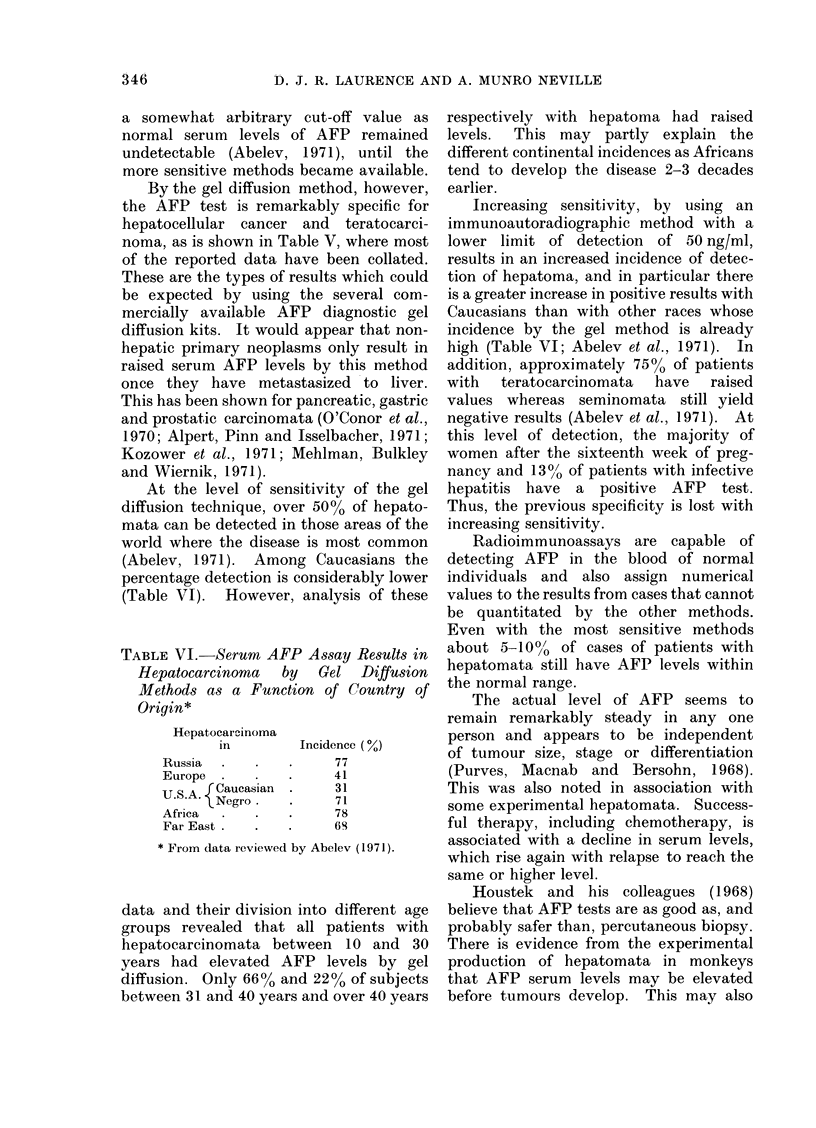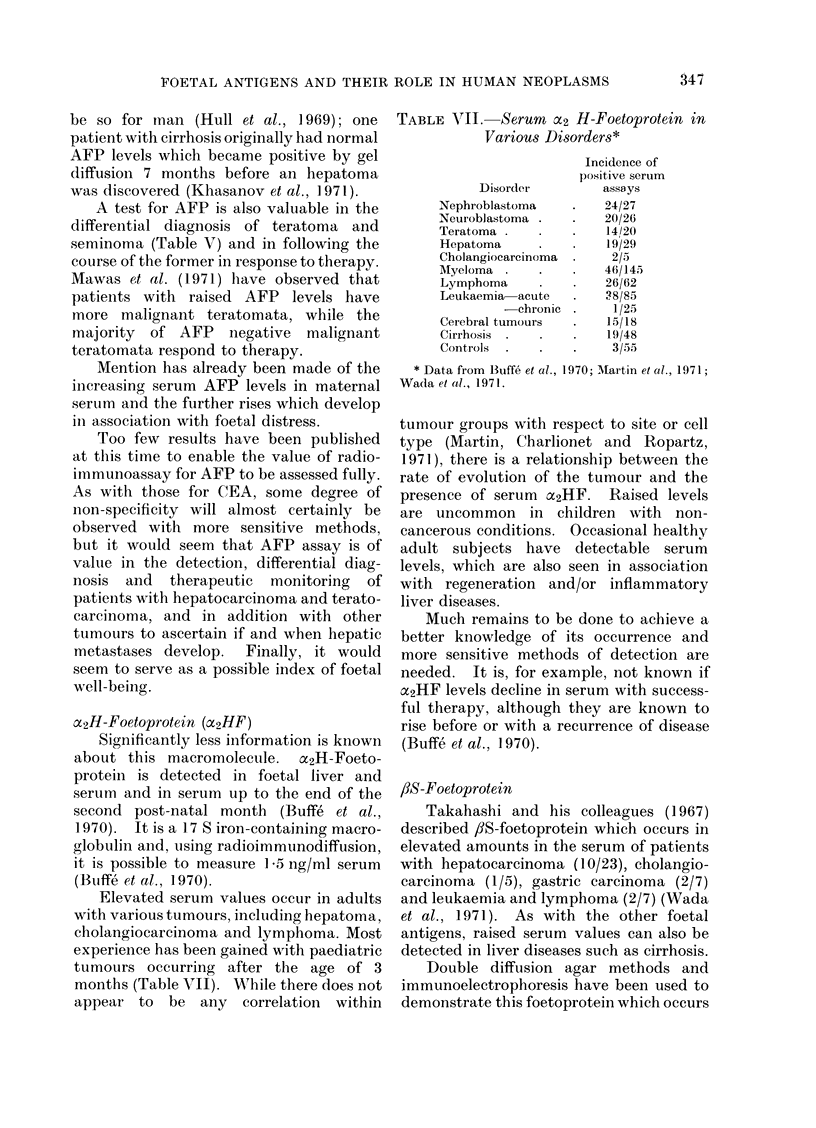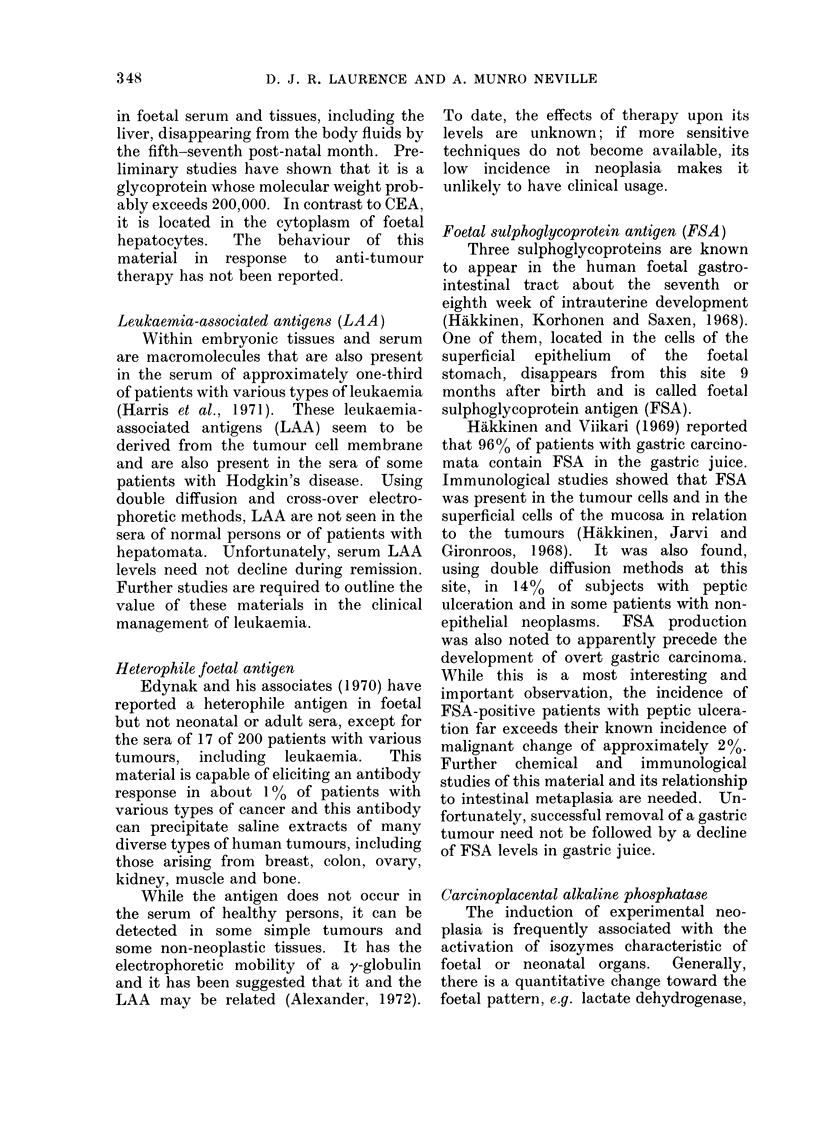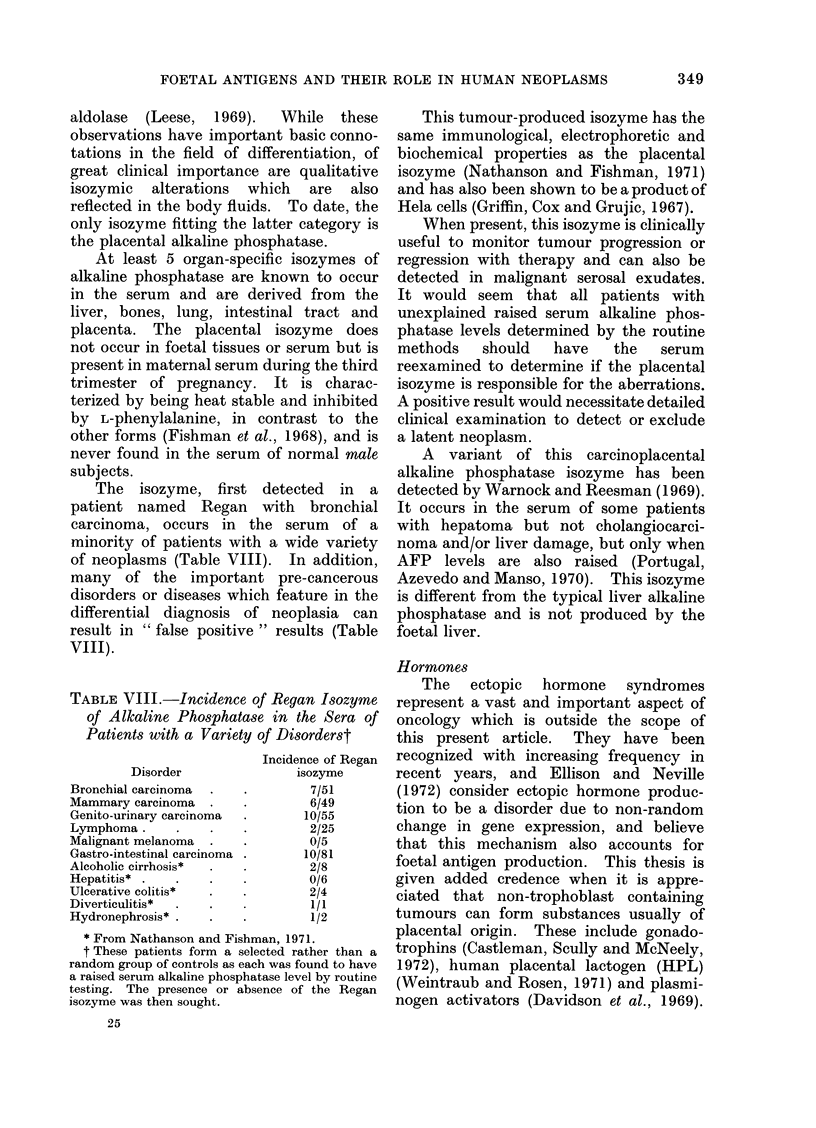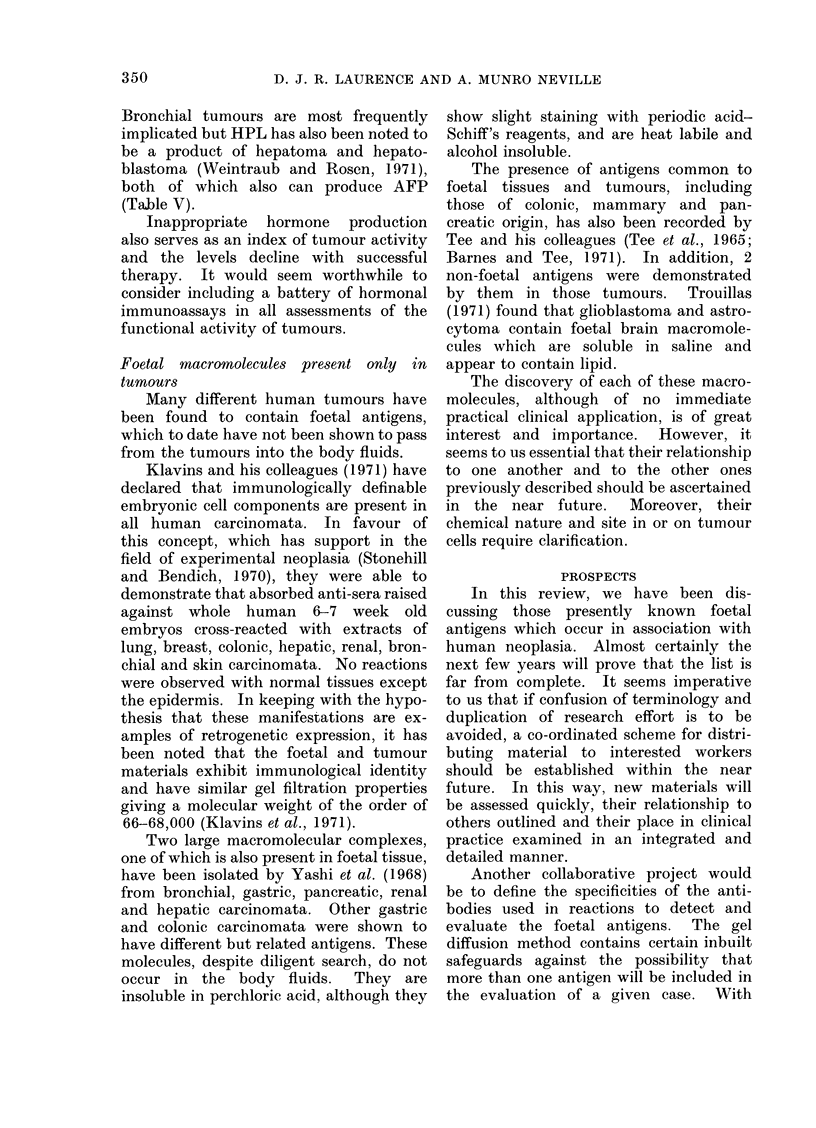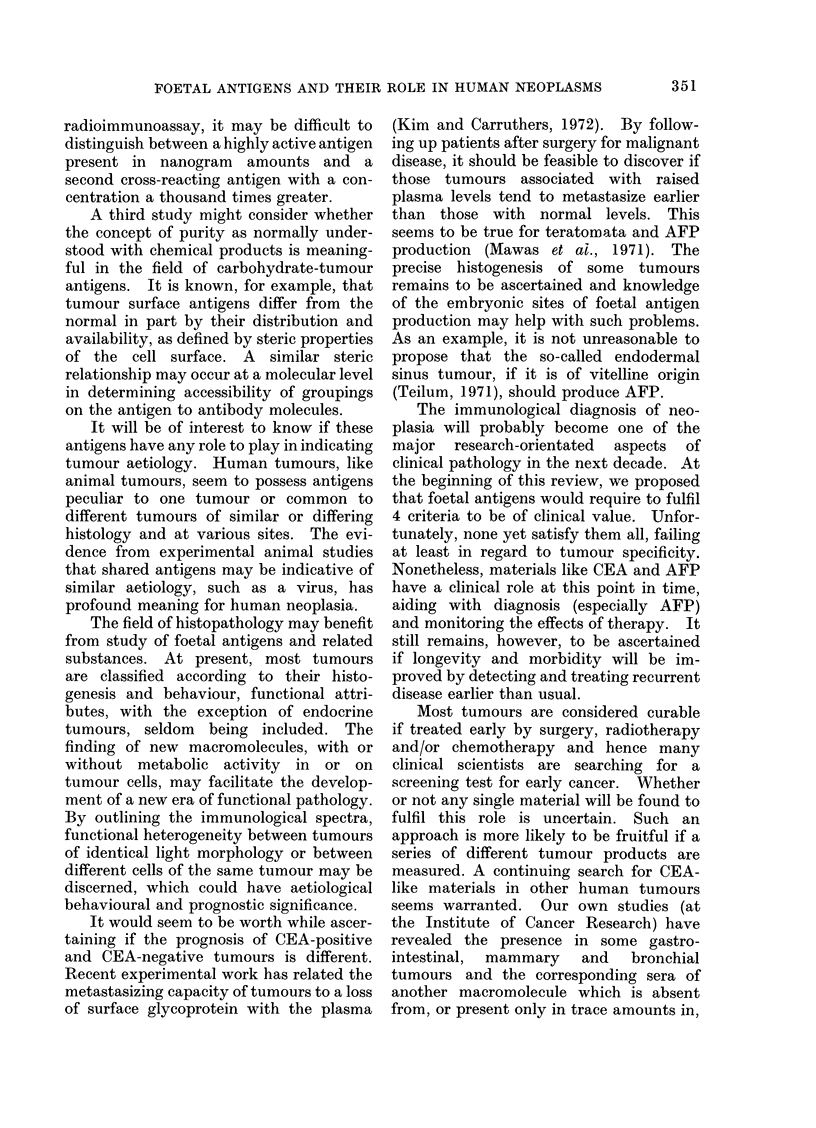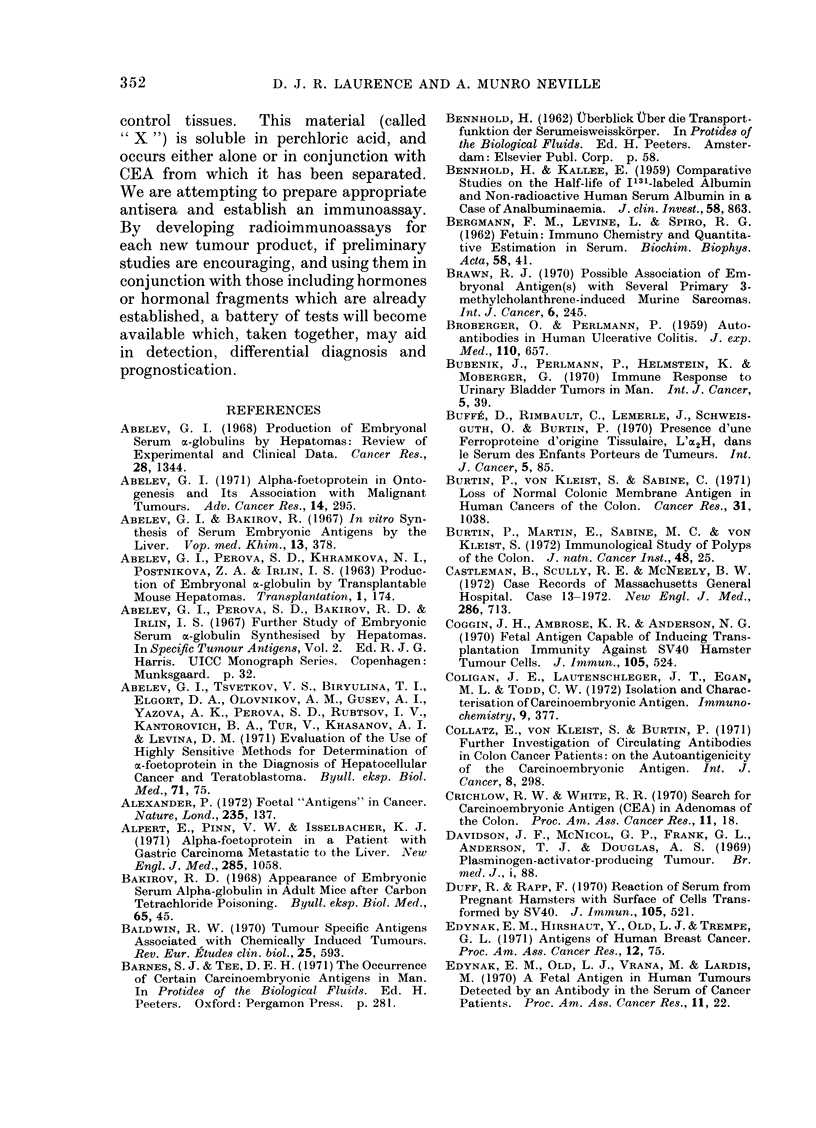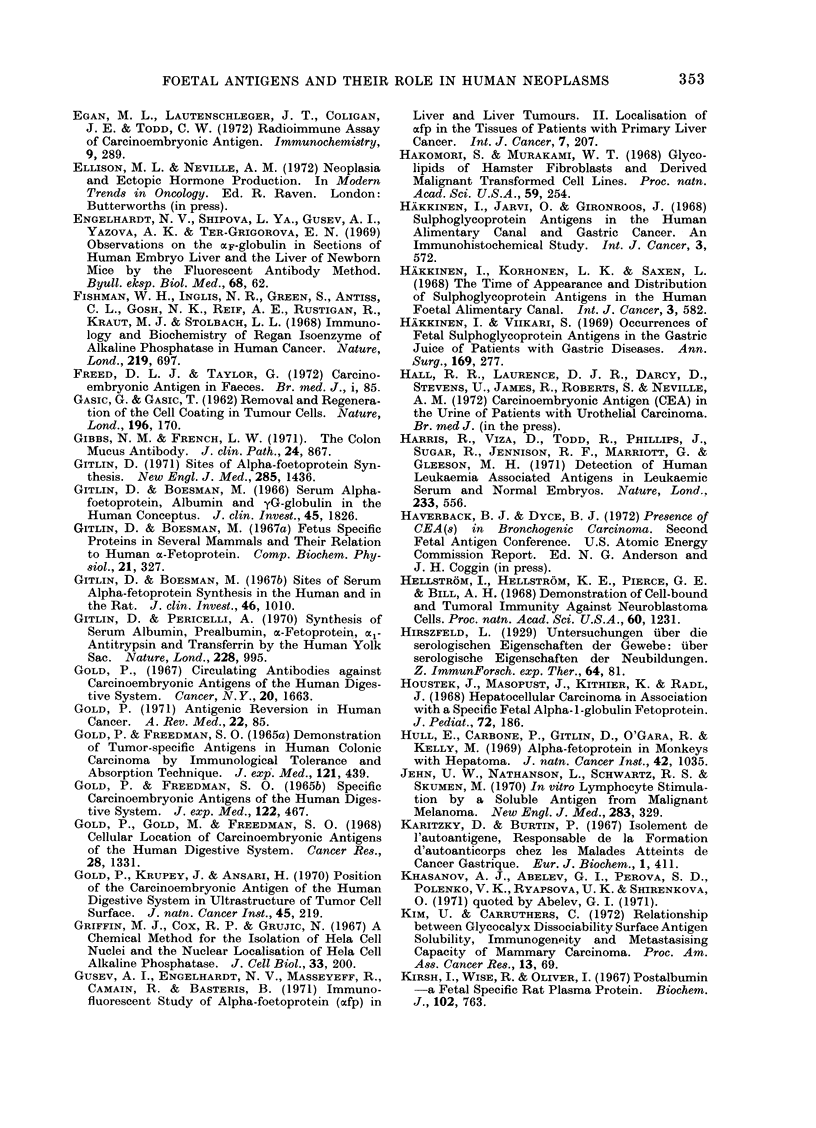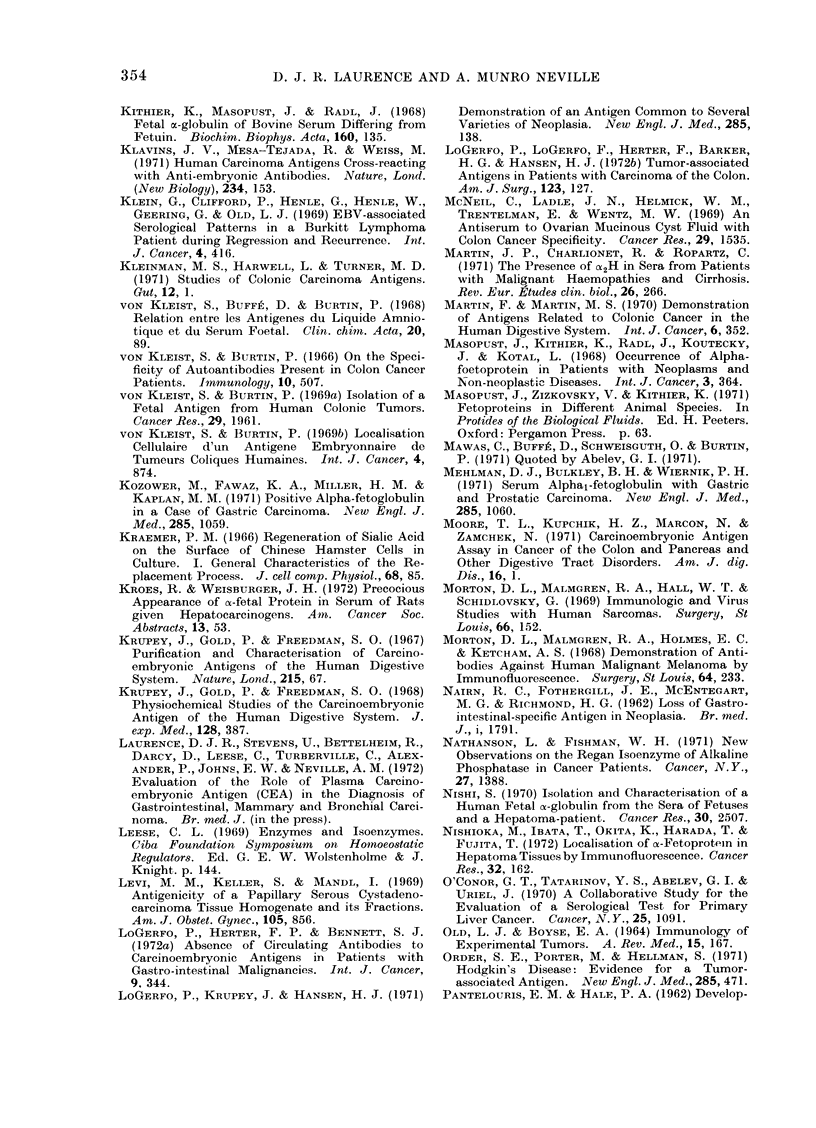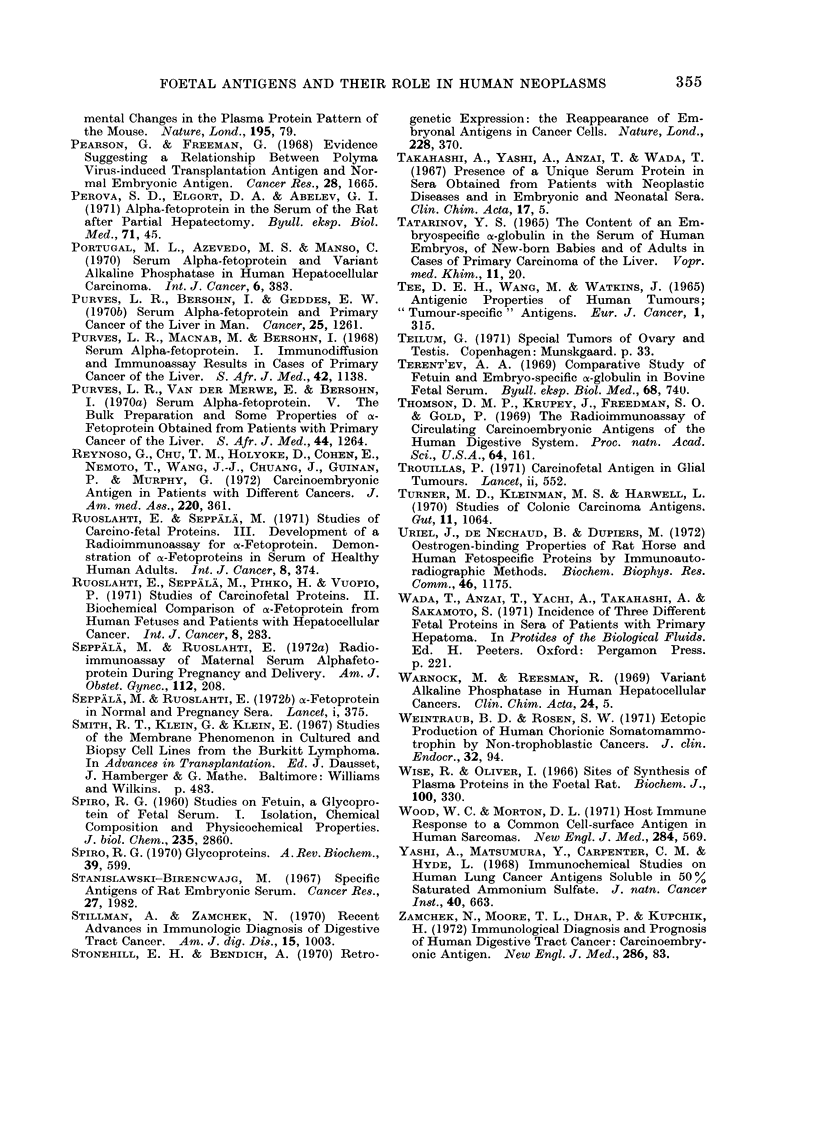# Foetal antigens and their role in the diagnosis and clinical management of human neoplasms: a review.

**DOI:** 10.1038/bjc.1972.45

**Published:** 1972-10

**Authors:** D. J. Laurence, A. M. Neville


*Br. J. Cancer* (1972) **26**, 335

### FOETAL ANTIGENS AND THEIR ROLE IN THE DIAGNOSIS AND CLINICAL MANAGEMENT OF HUMAN NEOPLASMS: A REVIEW

D. J. R. LAURENCE AND A. MUNRO NEVILLE

*From the Institute of Cancer Research, Royal Cancer Hospital, Chester Beatty Research Institute, Fulham Road, London, SW3 6JB*

Received 10 June 1972. Accepted 20 June 1972

DURING the past decade, there has been a renewed wave of interest in tumour immunology and in particular in the search for components or functions of cancer cells not shared by their normal counterparts. One goal has been to elucidate if tumours do or do not contain " tumour-specific or associated antigens ".

Most of the investigations have been effected using neoplasms of syngeneic animals and have clearly demonstrated in almost all tumours the existence of new antigens which are absent in presently detectable amounts from normal tissues (Old and Boyse, 1964; Baldwin, 1970; Stonehill and Bendich, 1970). While the syngeneic donor–host relationship seldom pertains in man, other experimental methods have clearly shown that many different types of human tumours, including colonic (Gold and Freedman, 1965*a*), ovarian (Levi, Keller and Mandl, 1969; McNeil *et al.*, 1969), bronchial (Yashi *et al.*, 1968), mammary (Edynak *et al.*, 1971) and urothelial carcinomata (Bubenik *et al.*, 1970), neuroblastoma (Hellström *et al.*, 1968), melanoma (Morton *et al.*, 1968; Jehn *et al.*, 1970), lymphomata (Smith, Klein and Klein, 1967; Klein *et al.*, 1969, Buffé *et al.*, 1970; Order, Porter and Hellman, 1971), leukaemia (Harris *et al.*, 1971) and sarcomata (Morton *et al.*, 1969; Wood and Morton, 1971), have tumour-associated antigens.

Of great interest has been the discovery of one particular group of materials, namely " embryo-specific or foetal antigens " in association with human (Gold and Freedman, 1965*a*; Tee, Wang and Watkins, 1965; Yashi *et al.*, 1968; Häkkinen and Viikari, 1969; Abelev, 1971; Klavins, Mesa-Tejada and Weiss, 1971; Trouillas, 1971) and animal tumours (Pearson and Freeman, 1968; Brawn, 1970; Coggin, Ambrose and Anderson, 1970; Duff and Rapp, 1970; Stonehill and Bendich, 1970; Alexander, 1972). The reappearance of embryo-specific materials was first postulated in human neoplasms as long ago as 1929 (Hirszfeld) and was noted by Stonehill and Bendich (1970) in diverse tumours of the mouse, rat and hamster induced by physical, viral and chemical agents, as well as those occurring spontaneously. Stonehill and Bendich (1970) proposed that this was a universal oncological phenomenon which they termed " retrogenetic expression ".

The observation that foetal antigens could be released into the body fluids from human tumours (Abelev, 1968; Thomson *et al.*, 1969) created a further dimension in that such materials could perhaps be of clinical value in the diagnosis and management of patients. To be clinically useful, these macromolecules would appear to require to fulfil 4 criteria: (1) pass from the tumour into the body fluids; (2) aid with the differential diagnosis of tumours by being found only with tumours *i.e.* cancer specific and if possible, site or tumour-type specific; (3) decline in amounts with successful therapy and rise again with recurrences; (4) be readily measurable by methods applicable for routine laboratory use.

The purpose of this present review is to discuss the association of foetal antigens and *human* neoplasms and examine their status in the diagnosis and management of tumour-bearing patients. In addition, attempts will be made to indicate those aspects which the authors consider are worthy of further intensive study or which require clarification before they are applied to the clinical situation.

Human tumour-associated foetal macromolecules (antigens) fall into 2 principal categories, depending upon whether or not they pass from the tumour cells into the plasma and other body fluids (Table I). Those which occur outside the tumour may be further subdivided according to the presence or absence of biological activity. Most of the article will be devoted to discussing the carcinoembryonic antigen (CEA) and $\alpha_1$-foetoprotein (AFP) which are the 2 materials whose role has been best elucidated.

TABLE I.—*Human Tumour-Associated Foetal Macromolecules (Antigens)*

1. Present in tumours and body fluids
   a. without known metabolic effects
      - Carcinoembryonic antigen (CEA).
      - $\alpha_1$-Foetoprotein (AFP).
      - $\alpha_2$H-Foetoprotein.
      - $\beta$S-Foetoprotein.
      - Leukaemia-associated antigens (LAA).
      - Heterophile foetal antigen.
      - Foetal sulphoglycoprotein antigen (FSA).
   b. with metabolic effects.
      - Placental alkaline phosphatase.
      - Placental-type hormones and related products.
2. Present in tumours only.

### *The carcinoembryonic antigen (CEA)*

Gold and Freedman (1965*a*) selected colonic adenocarcinomata as source of their human tumour antigen, hoping to minimize the difficulties of previous investigators in obtaining an autologous normal control tissue by employing normal mucosa taken at operation from a segment of the colon distal to the tumour itself. Rabbits were injected with pooled saline extracts of the adenocarcinomata and the resulting antisera, after absorption with saline extracts of the corresponding mucosa, human plasma, fibrin and killed gut bacteria, were reacted with saline tumour extracts to give a single line by double diffusion methods. Extracts of normal mucosa gave no reaction.

(a) *Tissue distribution.*—The distribution of the tumour antigen in a variety of human tumours and normal tissues was studied by the double diffusion precipitin technique. Twelve colonic carcinomata from different patients gave lines of complete identity with each other. Other tissues were tested and the tumour antigen was found in primary alimentary tract neoplasms from the oesophagus to the rectum, including the pancreas, together with their metastases. Primary tumours at other sites, secondary tumours of the alimentary tract and normal adult tissues were all negative (Gold and Freedman, 1965*b*). However, the alimentary tract, pancreas and liver of human foetuses aged between 2 and 6 months contained small amounts of an immunologically identical substance which, defined by these immunological tests, was called the " carcinoembryonic antigen ".

Many further studies have now been conducted confirming these original observations (von Kleist and Burtin, 1969*a* and *b*) and extending them to show the existence of material probably identical with CEA in some (Burtin *et al.*, 1972) but not all (Crichlow and White, 1970) colonic polyps, some inflammatory gastrointestinal and colonic disorders (Martin and Martin, 1970) and in bronchial carcinoma (Haverback and Dyce, 1972). With the development of radioimmunoassays for its measurement (Thomson *et al.*, 1969), CEA-reactive materials have been found to be present in the serum of normal healthy adults and in patients with a wide variety of inflammatory or regenerative disorders and benign and malignant neoplasms at many different sites (*vide infra*). The faeces of healthy subjects and of patients with colonic tumours also contain CEA-like substances (Freed and Taylor,

1972). In addition, the urine of patients with urothelial carcinoma or urinary tract infection contains substance(s) which interact with anti-CEA antibodies (Hall *et al.*, 1972).

(b) *Properties.*—Gold and Freedman (1965*a*) found that their antigen (CEA) had an electrophoretic mobility in agar gel similar to plasma $\beta$-globulin and was soluble in 0·6 mol/l perchloric acid, suggesting that it was a mucoprotein. Subsequent studies have indeed shown that CEA is a water-soluble glycoprotein, consisting predominantly of carbohydrate residues, including galactose, mannose, glucose, fucose, glucosamine and a variable amount of sialic acid (Krupey, Gold and Freedman, 1967, 1968). The presence of mannose and absence of N-acetylgalactosamine seem to distinguish it from blood group substances.

The amino acid composition of CEA was reported by Krupey *et al.* (1967, 1968) and some of our own recent work has also shown that the amino acids of blood group substance H and CEA are different.

It is unlikely that CEA is a single homogeneous material. More than one CEA peak has been eluted by gel filtration (Coligan *et al.*, 1972) or seen after ultracentrifugation (von Kleist and Burtin, 1969*a*).

In such sedimentation velocity experiments with purified CEA, Krupey *et al.* (1968) found a single peak with material isolated from the hepatic metastasis of a colonic tumour while a product from a hepatocarcinoma gave 3 peaks. Von Kleist and Burtin (1969*a*) and Coligan *et al.* (1972) have observed 2 ultracentrifuge peaks with products derived from colonic tumours. The faster sedimenting component may be a dimer of the slower component (von Kleist and Burtin, 1969*a*).

A number of authors have reported the antigenic complexity of CEA. Gold and Freedman (1965*a*) obtained 6 lines in double diffusion between colonic tumour extracts and antisera to colonic tumours in rabbits incompletely tolerant to normal mucosal extracts. Two of these lines were also given by normal mucosa. Of the remainder, 2 could be absorbed by extracts of normal mucosa while the other pair were both antigenically related to CEA. As noted above, the CEA isolated from a hepatoma which had 3 peaks in the ultracentrifuge (Krupey *et al.*, 1968) gave 2 lines against anti-CEA antiserum.

Turner (1970) and his colleagues (Turner, Kleinman and Harwell, 1970; Kleinman, Harwell and Turner, 1971) developed their own antisera in rabbits against crude perchloric acid extracts of colonic tumour proteins. The antisera, after absorbing with human AB cells, normal colonic mucosal extracts and human serum, gave 2 lines against the perchloric acid extracted colonic material. It was not possible to separate either antibodies or antigens responsible for the 2 precipitin lines, so they may have both been related to CEA. It was found that some of Gold's antisera also gave 2 lines against the Kleinman *et al.* (1971) extracts.

We have injected 5 goats with $\mu$g amounts of purified CEA according to the prescription of Egan *et al.* (1972). One of these goats developed 2 antibodies, one reacting with CEA and another reacting with a substance found in crude perchloric acid extracts of colonic tumours (the so-called X-substance). The relation of " X " to CEA appears to be complex but they may both share an antigenic determinant.

(c) *Metabolic significance.*—Gold (Gold, Gold and Freedman, 1968; Gold, Krupey and Ansari, 1970) and von Kleist and Burtin (1969*a*) found CEA to be localized in the glycocalyx of the plasma membranes of colonic tumour cells, and in particular in that part of the membrane bordering on the lumen of the neoplastic acini (von Kleist and Burtin, 1969*a*).

Several studies have indicated that the acquisition of a tumour antigen is accompanied by loss of normal cytoplasmic and membrane antigens (Nairn *et al.*, 1962; Burtin, von Kleist and Sabine, 1971)

which suggests that a precursor-product relationship may exist between the tumour and normal antigens. Certainly, the area of loss of normal antigens in a tumour, using several sections, enclosed those fields in which CEA was demonstrable (Burtin *et al.*, 1971).

The metabolism of the glycocalyx has been studied by Gasic and Gasic (1962) and Kraemer (1966), among others, who found that the carbohydrate layer can be replaced with a half-life of about one day. If this is true for CEA, it would account for its occurrence in plasma. Recent experimental studies seem to corroborate this view. Kim and Carruthers (1972) examined 2 strains of mammary carcinoma differing in metastasizing potential and found an inverse relationship between tumour content of a glycocalyx antigen, not necessarily CEA, and its concentration in the serum. Moreover, as the surface glycoprotein declined in amount, the metastasizing potential increased.

Another factor which determines the plasma level of CEA may be its rate of clearance. After complete resection of a colonic tumour, plasma CEA levels fall to a baseline level in a period that may be between 2 and 18 days (Laurence *et al.*, 1972; LoGerfo *et al.*, 1972*b*).

Much more information is required on the detailed structure of the CEA molecule, including the microheterogeneity of its sugar units (Spiro, 1970), both between molecules and within a single peptide chain. The mannose and N-acetylglucosamine content of CEA relates the CEA molecule to the core unit described by Spiro (1970) as a common feature of several glycoproteins linked to asparagine. The absence of N-acetylgalactosamine would suggest a lack of relationship with the serine-linked glycoproteins which include the blood group glycoproteins.

The production of CEA is dependent on the sequential action of a number of glycosyl transferases. According to Hakomori and Murakami (1968) the tumour fails to complete its antigens, at least those of a glycolipid nature. If CEA is an incomplete normal antigen, the normal product should be degradable to CEA. This possibility must be borne in mind when interpreting results on the distribution of CEA in tissues where autolysis or chemical treatment could result in some loss of terminal carbohydrate residues.

(d) *Autoantibodies to CEA.*—CEA is highly immunogenic in the goat, as demonstrated by the low dose injection schedules of Egan *et al.* (1972). Antibodies against colonic antigens have been detected in patients with colonic tumours and with ulcerative colitis (Broberger and Perlmann, 1959; von Kleist and Burtin, 1966; Gibbs and French, 1971). It would therefore not be surprising if some of these antibodies were directed against the CEA molecule.

Gold (1967) found that 70% of patients with localized cancer of the digestive system had antibodies with haemagglutinating activity against human O cells coated with CEA. The sera of 16 out of 20 women in the first and second trimesters of pregnancy had haemagglutinating activity. There was also activity in postpartum maternal serum. Patients with conditions other than colonic cancer or pregnancy had no activity.

Results from other laboratories have failed to find auto-antibodies to CEA. Karitzky and Burtin (1967) found that the anti-colon antibodies in colonic cancer patients reacted with antigens present in the normal colon. Collatz, von Kleist and Burtin (1971) used the patients' auto-antibodies to isolate the colonic antigens with which they reacted. These antigens were not CEA.

LoGerfo, Herter and Bennett (1972*a*) searched for antibodies able to bind labelled CEA in colonic tumour patients' plasma. The mixture of CEA and plasma was fractionated to identify CEA bound to macromolecules but none could be found. LoGerfo *et al.* (1972*a*) suggested that the Gold (1967) reaction was possibly due to blood group substances in his CEA that he used to coat the red cells. This explanation does not by itself account for the failure of the clinical control groups to show activity in Gold's experiments.

The assay of Egan *et al.* (1972) is potentially capable of registering CEA or anti-CEA in the plasma as " immuno-assayable CEA ". We have found that this assay separates certain West Indians of African origin into 2 groups with high (>20 ng/ml) plasma CEA (7 individuals) and normal (<10 ng/ml) CEA (3 individuals) (Laurence *et al.*, 1972). The high group all have anti-A isoantibodies in their plasma whereas the low group have no anti-A. No such effect can be detected in Caucasians or in a small number of Indians or Chinese.

There is no evidence that the high values with Africans are directly due to antibody binding; the anti-A activity may be correlated with presence of cross-reacting antigens in the plasma.

(e) *The CEA tests.*—Thomson *et al.* (1969) developed a radioimmunoassay which could detect 2·5 ng CEA/ml and found that all except one of 36 patients with active colonic or rectal cancer had raised serum levels which became undetectable after successful surgery. Of about 30 cases with digestive system cancer at other parts of the tract, only one (a disseminated pancreatic cancer) had a high CEA level; tumours outside the digestive system and non-neoplastic diseases also gave negative results.

Gold (1971) concluded that the CEA test was virtually diagnostic of digestive system cancer and with this specificity and high positive rate was a suitable test for screening.

Other groups of workers interested in the CEA concept developed radioimmunoassays (LoGerfo, Krupey and Hansen, 1971; Moore *et al.*, 1971; Egan *et al.*, 1972) of which the methods of LoGerfo *et al.* (1971) and Egan *et al.* (1972) have received most study. We have compared both methods, which employ different preparative methods and assay techniques. While 2·5 ng/ml is the upper limit of normal by one system (LoGerfo *et al.*, 1971), the comparable level in our hands using the other is 12·5 ng/ml. This is due to the presence of interfering background substances in the unextracted plasma used in the latter test system. We found that there was, however, a high degree of qualitative correlation between them (Laurence *et al.*, 1972). This is also substantiated by the almost uniform agreement between different groups as to the incidence of positive and negative results in a wide variety of disorders (Tables II and III).

The assay of plasma CEA seems to have most clinical application in the diagnosis of carcinoma of the gastro-intestinal tract, pancreas and bronchus, approximately 70–90% of which will yield raised levels (Table II). It is also of value in the assessment of neuroblastomata (Reynoso *et al.*, 1972; Table III) and possibly testicular and mammary neoplasms (Tables II and III). Unfortunately, its estimation has little or no part at present to play in the diagnosis of tumours at other sites (Table III; Laurence *et al.*, 1972).

Neither the cell type nor the degree of structural differentiation of mammary, bronchial and gastro-intestinal carcinomata seem to influence the level of plasma CEA. Rather, the extent of tumour spread seems to be the principal factor (Laurence *et al.*, 1972). This has been corroborated by others and also seems to be applicable to tumours at other sites (Table IV).

It is possible to divide the plasma CEA levels into 3 groups using the Egan *et al.* (1972) assay, namely normal (<12·5 ng/ml), intermediate (12·5–40 ng/ml) and high (>40 ng/ml). Patients with benign and malignant tumours and with inflammatory or regenerative disorders may fall into either the normal or intermediate groups (Tables II and III); levels in excess of 40 ng/ml are virtually diagnostic of malignancy (Laurence *et al.*, 1972). While 30% of mammary, 45% of colonic and 60% of bronchial carcinomata which are still localized can give intermediate or high values, only 8, 12 and 17% respectively of such early tumours yield levels in

TABLE II.—*Plasma CEA Levels Using Different Methods in Gastro-intestinal, Bronchial and Mammary Disorders*

| Site | Disorder | Incidence of positive plasma CEA assays: Zamchek *et al.*, 1972 | LoGerfo *et al.*, 1972b* | Reynoso *et al.*, 1972 | Laurence *et al.*, 1972 | Total (% incidence) |
|---|---|---|---|---|---|---|
| | Healthy controls | 0/40 | 0/100 | 1/138 | 0/57 | 1/335 (0·3%) |
| Gastro-intestinal tract | Carcinoma of | | | | | |
| | Colon and rectum | 43/60 | 97/133 | 29/35 | 61/88 | 230/316 (73%) |
| | Pancreas | 23/26 | 12/12 | 3/3 | 10/11 | 48/52 (92%) |
| | Liver | 0/2 | 8/12 | 1/1 | 3/3 | 12/18 (67%) |
| | Other sites | 6/14 | 23/30 | 8/11 | 14/30 | 51/85 (60%) |
| | Polyps of | | | | | |
| | Colon and rectum | 1/15 | 3/30 | 0/9 | 2/13 | 6/67 (9%) |
| | Inflammatory/Reactive | | | | | |
| | Ulcerative colitis and Crohn's disease | 1/41 | 6/13 | — | 13/41 | 20/95 (21%) |
| | Diverticulitis, peptic ulceration | — | 0/12 | — | 7/21 | 7/33 (21%) |
| | Cirrhosis and alcoholic liver disease | 24/46† | 0/15 | — | 4/5 | 28/66 (42%) |
| | Alcoholic pancreatitis | 17/32† | — | — | — | 17/32 (53%) |
| | Other | — | 1/19 | — | 1/24 | 2/43 (5%) |
| Breast | Carcinoma | 0/1 | 29/44 | 16/35 | 37/74 | 82/159 (52%) |
| | Benign tumour | — | — | 0/4 | 1/20 | 1/24 (4%) |
| | Reactive | | | | | |
| | Fibroadenosis | — | 0/16 | — | 5/54 | 5/70 (7%) |
| Respiratory tract | Carcinoma of | | | | | |
| | Bronchus | 6/8 | 26/35 | 7/10 | 26/37 | 65/90 (72%) |
| | Upper respiratory tract | 0/1 | — | — | 8/14 | 8/15 (53%) |
| | Inflammatory/Reactive | | | | | |
| | Pulmonary tuberculosis | — | — | — | 8/21 | 8/21 (38%) |
| | Chronic bronchitis and emphysema | — | 5/42 | — | 11/21 | 16/63 (25%) |

* See also Moore *et al.*, 1971 and LoGerfo *et al.*, 1971.
† Non-alcoholic liver and pancreatic diseases gave normal values.

TABLE III.—*Plasma CEA Levels Using Different Methods in a Variety of Different Neoplastic and Reactive Disorders*

| Site | Disorder | Incidence of raised plasma CEA assays: Zamchek *et al.*, 1972 | LoGerfo *et al.*, 1972 | Reynoso *et al.*, 1972 | Laurence *et al.*, 1972 | Total (% incidence) |
|---|---|---|---|---|---|---|
| Uterus | Carcinoma of | | | | | |
| | Cervix | — | 3/6 | 7/19 | 9/20 | 19/45 (42%) |
| | Endometrium | — | — | 2/4 | 1/7 | 3/11 (27%) |
| Ovary | Carcinoma | — | 4/10 | 1/5 | 2/5 | 7/20 (35%) |
| | Benign tumours | — | — | — | 0/2 | 0/2 (0%) |
| Testis | Seminoma | — | — | 4/9 | 1/1 | 5/10 (50%) |
| | Teratocarcinoma | — | — | 6/12 | 5/7 | 11/19 (58%) |
| | Other | — | — | 1/3 | — | 1/3 (33%) |
| Prostate | Carcinoma | — | 25/52 | 8/32 | 4/9 | 37/93 (40%) |
| | Reactive hypertrophy | — | 0/17 | 1/11 | 1/2 | 2/30 (7%) |
| Kidney | Carcinoma | — | 2/8 | 4/7 | 3/11 | 9/26 (35%) |
| | Renal failure | 6/12 | 0/13 | — | — | 6/25 (24%) |
| Bladder | Carcinoma | — | 6/12 | 11/45 | 18/49 | 34/106 (33%) |
| Nervous system | Neuroblastoma | — | — | 6/6 | — | 6/6 (100%) |
| | Intracerebral malignant tumours | — | — | — | 2/7 | 2/7 (29%) |
| Haematological | Leukaemia | — | — | 2/20 | 4/7 | 6/27 (22%) |
| | Lymphoma | 0/2 | 2/18 | 1/7 | 11/29 | 14/56 (25%) |

TABLE IV.—*Influence of Stage of Tumour Progression on the Plasma CEA Levels*

| Carcinoma of | Stage | Reynoso *et al.*, 1972 | Laurence *et al.*, 1972 | Total (% incidence) |
|---|---|---|---|---|
| Colon and rectum | Dukes A | 7/19* | 13/29 | 20/48 (42%) |
| | Dukes B | 14/23* | 22/29 | 36/52 (69%) |
| | Dukes C | 21/28* | 6/10 | 27/38 (71%) |
| | Metastasized | 46/54* | 20/20 | 66/74 (89%) |
| Bronchus | Local NO, MO | — | 15/24 | 15/24 (63%) |
| | Local N+, MO | — | 5/6 | 5/6 (83%) |
| | Metastases | — | 6/7 | 6/7 (86%) |
| Breast | Local NO, MO | 1/10 | 12/39 | 22/69 (32%) |
| | Local N+, MO | | 9/20 | |
| | Metastases | 15/25 | 6/7 | 21/32 (66%) |
| Prostate | Local | 4/24 | 1/4 | 5/28 (18%) |
| | Metastases | 4/8 | 3/5 | 7/13 (54%) |
| Bladder | Local | 9/35 | 13/34 | 22/69 (32%) |
| | Metastases | 2/11 | 6/13 | 8/24 (33%) |
| Cervix | Stage 0 | 0/4 | 1/3 | 1/7 (14%) |
| | Stage 1 | 2/5 | 3/9 | 5/14 (36%) |
| | Stage 2 | 3/5 | 2/4 | 5/9 (55%) |
| | Stage 3 | 2/5 | 3/4 | 5/9 (55%) |

* LoGerfo *et al.*, 1972*b*.

the " cancer " diagnostic range (>40 ng/ml). Hence, when it is appreciated that each of these cases was a clinically overt neoplasm, the detection rate of even earlier lesions may be less. Thus, at present estimation of plasma CEA levels does not appear to be a worthwhile screening procedure. However, we are unable to state that by detecting a group of subjects with raised plasma CEA levels and then investigating them in detail, lives would not be saved. In fact, some patients have had high CEA levels before overt neoplasia develops (Stillman and Zamchek, 1970).

Disorders such as diverticulitis, peptic ulceration, ulcerative colitis and Crohn's disease, which feature prominently in the differential diagnosis of gastrointestinal neoplasia, can give CEA levels in the intermediate (12·5–40 ng/ml) range (Table II; Laurence *et al.*, 1972). Detectable carcinomata were not found in any of the subjects in our series, and with ulcerative colitis and Crohn's disease there did not appear to be any correlation with the disease severity. Consequently, CEA has little value, if any, in the differential diagnosis of gastro-intestinal neoplasia. Similar conclusions may be drawn for pulmonary disease. The highest incidence of " false positive values " occurs in patients with chronic bronchitis and emphysema and those who also smoke cigarettes, the very group in which it would have been valuable to use the test to screen for bronchial neoplasia. However, it may be premature to dismiss the raised values in patients with other disorders as " false positives " in view of the observations of Stillman and Zamchek (1970).

The most valuable aspect of the CEA test at present would appear to be as an adjunct to monitor therapy and to detect residual disease and the development of metastases. If the plasma CEA is raised pre-operatively, it declines to normal between the second and the eighteenth post-operative day, if the tumour has been completely removed. A remaining high level indicates residual disease. From limited experience, the levels almost always rise again with the development of metastases (Table IV; Laurence *et al.*, 1972), and it would appear worth while now to assay CEA pre-operatively and during the post-operative phase and at each out-patient attendance, to ascertain if the presence of recurrent or residual disease may be detected earlier. Whether this will affect the long-term survival or prognosis remains to be determined.

The estimation of CEA in urine in patients with bladder carcinomata has also clinical and diagnostic significance. High levels of CEA-like materials can be detected in approximately 70% of patients with bladder tumours and even in those with early *in situ* lesions. Patients with urinary infection, however, may give falsely elevated levels which can also arise from contamination with vaginal or cervical secretions (Hall *et al.*, 1972).

Successful removal of the bladder tumour is associated with a decline to normal levels. Of interest is that hypernephromata and other non-urothelial tumours do not cause a raised urinary CEA level even in the presence of high plasma levels, except when such tumours invade the urothelial passage.

*The alpha-foetoprotein (AFT)*

In 1963, Abelev *et al.* developed antisera against the serum proteins of newborn mice and absorbed them with adult mouse serum. The resulting absorbed antisera reacted not only with a component of newborn mouse serum but also with the sera of adult mice carrying a transplantable hepatoma. The foetal serum protein, which had the mobility of an $\alpha_1$-globulin, was also detected in the blood of adult mice after partial hepatectomy and of adult mice carrying 2 other transplantable hepatomata. No antigen was present in the serum of mice with transplantable tumours other than hepatomata.

Tatarinov (1965) observed reaction between an antigen in the blood of 2 patients with primary carcinoma of the liver and the monospecific antiserum against a foetal component of human blood.

Other workers (Kithier, Masopust and Radl, 1968; Terent'ev, 1969) have shown that foetal calf serum contains several distinct foetal proteins, one of which is fetuin, which is not increased in cows with hepatocellular carcinoma whereas a second $\alpha_1$-globulin component is elevated. The occurrence of a distinct protein species that is predominant in early foetal serum and also hepatoma-related has been established in 18 mammalian species by immunological cross reactions (Gitlin and Boesmann, 1967*a*). This component is now given the general name, alpha-foetoprotein (AFP).

(a) *Tissue distribution.*—Studies on the distribution of AFP have been conditioned by the detection method. In early foetal serum and in the serum of certain patients with hepatomata, the protein band is easily visualized by zone electrophoresis which can detect 300 μg/ml AFP in serum. Double diffusion, which has been the principal detection method until 1970, has a sensitivity of 1–3 μg/ml. More recently (Abelev *et al.*, 1971) aggregate-haemagglutination and immunoautoradiography have detected 50 ng/ml while radioimmunoassay can respond to 0·25 ng/ml (Ruoslahti and Seppälä, 1971).

With development of more sensitive methods, the somewhat capricious differences found in early work, *e.g.* between mice and rats or between Caucasians and Africans, have been shown to be quantitative rather than qualitative in nature. AFP is the major protein component in the serum of early rodent (Pantelouris and Hale, 1962; Kirsh, Wise and Oliver, 1967) and human foetuses; the highest level in the human foetus is of the order of 3–4 mg/ml serum (Gitlin and Boesman, 1966) and is attained around the thirteenth week of intrauterine development, having been detected by double diffusion technique from approximately the sixth week. With increasing foetal age, AFP levels decline and albumin concentrations rise, and by the end of the first post-natal week, AFP is no longer demonstrable. The transition between AFP and albumin, as the predominant foetal serum protein, occurs somewhat later in relation to gestational age in rodents than in man. The cut-off point at which AFP is no longer detectable by double diffusion methods is also later in rodents (Tatarinov, 1965). However, the use of sensitive radioimmunoassay has shown that small amounts (4–25 ng/ml) persist in normal adult serum (Ruoslahti and Seppälä, 1971). In addition, trace amounts are detected in cord blood and in the amniotic fluid (von Kleist *et al.*, 1968). Recent results have also shown that the AFP content of sera of pregnant women departs from the normal value (Abelev *et al.*, 1971; Seppälä and Ruoslahti, 1972*a*). During the first, second and third trimesters, the maternal serum levels vary between 18 and 119 ng/ml, 96 and 302 ng/ml and 103 and 550 ng/ml respectively, dropping shortly before term to normal. Levels in excess of 1000 ng/ml occur before and after foetal death, and it has been postulated that an increased release of AFP may occur because of foetal distress (Seppälä and Ruoslahti, 1972*b*).

AFP is formed principally by the rodent and human foetal liver, but also by the yolk sac and gastro-intestinal tract (Gitlin and Boesman, 1967*b*; Engelhardt *et al.*, 1969; Gitlin and Pericelli, 1970; Gitlin, 1971). Other foetal tissues, including spleen, lung and kidney, the placenta and adult liver do not appear to form AFP in significant amounts (Wise and Oliver, 1966; Abelev and Bakirov, 1967). Of further interest are the reports which showed that its major synthetic site in sharks is the gastro-intestinal tract and, in birds, the yolk sac (*e.g.*, Gitlin, 1971).

In the liver, most hepatocytes are initially involved in AFP synthesis by the sixth week of foetal life, but as the serum levels commence to fall the numbers of cells staining immunofluorescently decline also and tend to be those situated around the central vein. The Kupffer cells, bile duct epithelium and haemopoietic cells do not appear to synthesize AFP at any time (Engelhardt *et al.*, 1969).

In regenerating liver (Engelhardt *et al.*, 1969) and also in hepatoma (Gusev *et al.*, 1971) only certain cells in the tissue give an AFP reaction by immunofluorescence. The " positive " hepatoma cells were more frequently adjacent to the tumour vessels, suggesting that production of AFP is not entirely a random process. Nishioka *et al.* (1972) have observed that less than 20% of the cells of a hepatoma contained AFP. The AFP-containing cells had larger, more hyperchromic nuclei than the AFP negative cells.

(b) *Properties.*—AFP belongs to a class of proteins widely distributed among mammalian species (Gitlin and Boesman, 1967*a*; Masopust, Zizkovsky and Kithier, 1971). AFP of human origin is an $\alpha_1$-globulin with a molecular weight of 64,000 and a sedimentation constant of 4·5 (Nishi, 1970). Approximately 4% of the total protein is carbohydrate (Ruoslahti *et al.*, 1971). Hexose, hexosamine and sialic acid occur in it in a ratio of 2·2 : 1·2 : 0·9 by weight. While samples of the proteins from foetal sources show uniform electrophoretic mobilities those of tumour origin have up to 4 variants or sub-components with slightly different mobilities (Purves, Van de Merwe and Bersohn, 1970*a*). By treating with neuroaminidase and removing the sialic acid from the carbohydrate component of the protein, these differences between the variant tumour products are removed so that such differences between products are probably related to variations in the carbohydrate rather than the peptide changes.

Nishi (1970) and Ruoslahti and his colleagues (1971) have determined the amino acid composition of AFP after purification using immunological methods. The results of the 2 groups agree very well. Each finds that AFP isolated from foetal serum and from serum of a hepatoma patient have very similar total amino acid compositions. The latter group have also found identical peptide maps for tumour and foetal AFP.

Abelev (1971) has emphasized the similarity in chemical and physical properties of albumin and AFP, which makes separation by other than immunological methods difficult. The amino acid compositions of AFP and albumin are very similar. However, the content of glycine and of isoleucine in human albumin is about half and one-third respectively that of AFP. The serine and threonine content of AFP is about 50% higher than in albumin. Both AFP and albumin have a high leucine content (about 10% of all amino acid residues). Like albumin, AFP can bind oestrogens but AFP cannot bind testosterone (Uriel, de Nechaud and Dupiers, 1972), though this steroid is strongly bound to albumin.

AFP is clearly distinct from fetuin in its carbohydrate—fetuin has 20% carbohydrate—and amino acid composition (Spiro, 1960). Their solubility characteristics and immunological properties are also widely different (Bergmann, Levine and Spiro, 1962; Kithier *et al.*, 1968; Terent'ev, 1969).

*Metabolic significance*

The role played by AFP in foetal life is not clear; it appears to be a substitute for albumin of the adult serum. As some adult individuals lacking serum albumin (Bennhold and Kallee, 1959) seem to be able to live without gross metabolic defects, the similarity of albumin to AFP does not help to determine the true function of the latter. Certainly, albumin plays an important part in controlling haemolytic jaundice of the newborn (Bennhold, 1962) and a similar "emergency overflow" mechanism may operate for AFP in the foetus. AFP is known to bind oestrogens (Uriel *et al.*, 1972).

The recent development of radioimmunoassay techniques for AFP resulted in its demonstration in the serum of healthy adults so that, like CEA, there does not appear to be complete gene suppression as was originally considered from the data of less sensitive methods. Hence, reappearance of AFP in liver diseases of the infant (Masopust *et al.* 1968, 1971) and adult associated with inflammation and/or regeneration (Abelev *et al.*, 1963; Table V; Abelev, 1971) is not unexpected. However, the explanation is not a simple one. Abelev *et al.* (1967) pointed out that when AFP levels are falling in late gestation, the liver is still undergoing very rapid proliferation, and that this rapid growth continues into early childhood when no AFP can be found in the serum by gel diffusion.

In experimental animals, acute poisoning with carbon tetrachloride leads to a rise in AFP level; a peak develops at 2–3 days after exposure, declining thereafter and becoming negative 8–10 days later

TABLE V.—*Serum AFP Assay Results by Gel Diffusion Methods*

| Site | Disorders | Incidence of positive serum AFP assays: Number | % |
|---|---|---|---|
| | Normal controls | 0/15,730 | (0) |
| | Pregnancy | 13/1069 | (1·2) |
| Liver | Hepatocarcinoma | 589/868 | (68) |
| | Cholangiocarcinoma | 1/73 | (1·4) |
| | Non-neoplastic* | 15/6484 | (0·2) |
| Gonad | Teratocarcinoma | 49/108 | (45) |
| | Seminoma | 0/39 | (0) |
| | Choriocarcinoma | 2/15 | (13) |
| | Other tumours | 0/13 | (0) |
| Kidney | Nephroblastoma | 0/59 | (0) |
| | Neuroblastoma | 0/70 | (0) |
| | Non-hepatic primary malignant tumours† | 10/1169 | (0·8) |
| | Miscellaneous‡ | 1/3373 | (0·03) |

* Twelve of the positive assays had either viral hepatitis or cirrhosis.
† All 10 positive assays were in patients with hepatic metastases.
‡ The positive is an example of hepatoblastoma.

From the data reviewed by Abelev, 1971; Masopust *et al.*, 1971; Kozower *et al.*, 1971; Mehlman *et al.*, 1971; Alpert *et al.*, 1971.

(Bakirov, 1968; Perova, Elgort and Abelev, 1971). The experiment may then be repeated on the same animals with similar results.

Hepatomata induced in rats by a variety of carcinogens gave different incidences of raised serum AFP levels (Stanislawski–Birencwajg, 1967) depending on the inducing agent and the manner of its application. However, aflatoxin-induced tumours were consistently negative.

While Abelev *et al.* (1967) have found a correlation between AFP levels and the time required for transplantable hepatomata to kill the host animal, no such correlation has been found for tumour subjects though Purves, Bersohn and Geddes (1970*b*) found lower serum levels in patients with the more differentiated tumours.

There is a wide range of AFP levels in patients with hepatomata. Although the total range between patients is 500,000-fold, the variation for a given patient is rarely more than 10-fold over the time of observation (Purves *et al.*, 1970*b*). As the outcome of the disease does not appear to differ depending on the AFP level, this suggests that there is a homoeostatic control mechanism for AFP production in the tumour cells. This does not seem to be true for teratocarcinomata (Mawas *et al.*, 1971).

The time course of AFP production is little understood. Hull *et al.* (1969) have shown that AFP production can precede the development of nitrosodiethylamine-induced hepatomata in monkeys. In others there is no apparent AFP production. Such tumours possess a prominent lymphocytic infiltrate, which disappears if and when these lesions start to form AFP. Kroes and Weisburger (1972) observed a burst of AFP production in rats 3–5 weeks after administering 3-methyl DAB together with aflatoxin. The AFP level returned to normal until the tumour developed, when a second increase in serum AFP concentration occurred.

At a cellular level, the presence of AFP in only a few of the tumour cells suggests that AFP production is an activity of a minority of the cell population. An alternative interpretation would be that all the cells are making AFP but few are storing the protein. If the AFP-producing cells are associated with the vessels as suggested by Gusev *et al.* (1971) then AFP production or the location of AFP cells must be subject to physiological gradients.

The presence of AFP in normal adults means that some cells, not visualized by immunofluorescence, continue to produce AFP throughout life. Either these are very few cells working at normal rate or a larger number of cells that are modulated to a subnormal rate. Restoration of AFP production would then be by proliferation of a very small number of cells, remodulation to a higher level of production, or perhaps both.

At a molecular level, the genetic information required to make the peptide portion of AFP would be expected to be present in the genome of adult cells. Expression of the genetic information would require a lifting of repression at the gene level, possibly together with the development of protein synthetic apparatus and the multiplication of committed cells. The synthesis and control of the carbohydrate chains of AFP, which are apparently the source of variation in the molecule (Purves *et al.*, 1970*a*), would be dependent less directly upon information in the genome. Like CEA, these carbohydrate chains are the result of sequential action of sugar transferases at appropriate cell loci. The variant chains could be the result of differences in relative concentrations and location of these enzymes in the cytoplasm.

(d) *The AFP tests.*—The history of the several AFP tests introduced during the past few years is one of progressive improvement in sensitivity over a 1000-fold range. The double diffusion technique, which was the most sensitive method up until 1970 and was capable of measuring 1 $\mu$g AFP/ml serum, introduced a somewhat arbitrary cut-off value as normal serum levels of AFP remained undetectable (Abelev, 1971), until the more sensitive methods became available.

By the gel diffusion method, however, the AFP test is remarkably specific for hepatocellular cancer and teratocarcinoma, as is shown in Table V, where most of the reported data have been collated. These are the types of results which could be expected by using the several commercially available AFP diagnostic gel diffusion kits. It would appear that non-hepatic primary neoplasms only result in raised serum AFP levels by this method once they have metastasized to liver. This has been shown for pancreatic, gastric and prostatic carcinomata (O'Conor *et al.*, 1970; Alpert, Pinn and Isselbacher, 1971; Kozower *et al.*, 1971; Mehlman, Bulkley and Wiernik, 1971).

At the level of sensitivity of the gel diffusion technique, over 50% of hepatomata can be detected in those areas of the world where the disease is most common (Abelev, 1971). Among Caucasians the percentage detection is considerably lower (Table VI). However, analysis of these

TABLE VI.—*Serum AFP Assay Results in Hepatocarcinoma by Gel Diffusion Methods as a Function of Country of Origin**

| Hepatocarcinoma in | | Incidence (%) |
|---|---|---|
| Russia | | 77 |
| Europe | | 41 |
| U.S.A. | Caucasian | 31 |
| | Negro | 71 |
| Africa | | 78 |
| Far East | | 68 |

* From data reviewed by Abelev (1971).

data and their division into different age groups revealed that all patients with hepatocarcinomata between 10 and 30 years had elevated AFP levels by gel diffusion. Only 66% and 22% of subjects between 31 and 40 years and over 40 years respectively with hepatoma had raised levels. This may partly explain the different continental incidences as Africans tend to develop the disease 2–3 decades earlier.

Increasing sensitivity, by using an immunoautoradiographic method with a lower limit of detection of 50 ng/ml, results in an increased incidence of detection of hepatoma, and in particular there is a greater increase in positive results with Caucasians than with other races whose incidence by the gel method is already high (Table VI; Abelev *et al.*, 1971). In addition, approximately 75% of patients with teratocarcinomata have raised values whereas seminomata still yield negative results (Abelev *et al.*, 1971). At this level of detection, the majority of women after the sixteenth week of pregnancy and 13% of patients with infective hepatitis have a positive AFP test. Thus, the previous specificity is lost with increasing sensitivity.

Radioimmunoassays are capable of detecting AFP in the blood of normal individuals and also assign numerical values to the results from cases that cannot be quantitated by the other methods. Even with the most sensitive methods about 5–10% of cases of patients with hepatomata still have AFP levels within the normal range.

The actual level of AFP seems to remain remarkably steady in any one person and appears to be independent of tumour size, stage or differentiation (Purves, Macnab and Bersohn, 1968). This was also noted in association with some experimental hepatomata. Successful therapy, including chemotherapy, is associated with a decline in serum levels, which rise again with relapse to reach the same or higher level.

Houstek and his colleagues (1968) believe that AFP tests are as good as, and probably safer than, percutaneous biopsy. There is evidence from the experimental production of hepatomata in monkeys that AFP serum levels may be elevated before tumours develop. This may also be so for man (Hull *et al.*, 1969); one patient with cirrhosis originally had normal AFP levels which became positive by gel diffusion 7 months before an hepatoma was discovered (Khasanov *et al.*, 1971).

A test for AFP is also valuable in the differential diagnosis of teratoma and seminoma (Table V) and in following the course of the former in response to therapy. Mawas *et al.* (1971) have observed that patients with raised AFP levels have more malignant teratomata, while the majority of AFP negative malignant teratomata respond to therapy.

Mention has already been made of the increasing serum AFP levels in maternal serum and the further rises which develop in association with foetal distress.

Too few results have been published at this time to enable the value of radioimmunoassay for AFP to be assessed fully. As with those for CEA, some degree of non-specificity will almost certainly be observed with more sensitive methods, but it would seem that AFP assay is of value in the detection, differential diagnosis and therapeutic monitoring of patients with hepatocarcinoma and teratocarcinoma, and in addition with other tumours to ascertain if and when hepatic metastases develop. Finally, it would seem to serve as a possible index of foetal well-being.

TABLE VII.—*Serum $\alpha_2$ H-Foetoprotein in Various Disorders**

| Disorder | Incidence of positive serum assays |
|---|---|
| Nephroblastoma | 24/27 |
| Neuroblastoma | 20/26 |
| Teratoma | 14/20 |
| Hepatoma | 19/29 |
| Cholangiocarcinoma | 2/5 |
| Myeloma | 46/145 |
| Lymphoma | 26/62 |
| Leukaemia—acute | 38/85 |
| —chronic | 1/25 |
| Cerebral tumours | 15/18 |
| Cirrhosis | 19/48 |
| Controls | 3/55 |

* Data from Buffé *et al.*, 1970; Martin *et al.*, 1971; Wada *et al.*, 1971.

*$\alpha_2$H-Foetoprotein ($\alpha_2$HF)*

Significantly less information is known about this macromolecule. $\alpha_2$H-Foetoprotein is detected in foetal liver and serum and in serum up to the end of the second post-natal month (Buffé *et al.*, 1970). It is a 17 S iron-containing macroglobulin and, using radioimmunodiffusion, it is possible to measure 1·5 ng/ml serum (Buffé *et al.*, 1970).

Elevated serum values occur in adults with various tumours, including hepatoma, cholangiocarcinoma and lymphoma. Most experience has been gained with paediatric tumours occurring after the age of 3 months (Table VII). While there does not appear to be any correlation within tumour groups with respect to site or cell type (Martin, Charlionet and Ropartz, 1971), there is a relationship between the rate of evolution of the tumour and the presence of serum $\alpha_2$HF. Raised levels are uncommon in children with non-cancerous conditions. Occasional healthy adult subjects have detectable serum levels, which are also seen in association with regeneration and/or inflammatory liver diseases.

Much remains to be done to achieve a better knowledge of its occurrence and more sensitive methods of detection are needed. It is, for example, not known if $\alpha_2$HF levels decline in serum with successful therapy, although they are known to rise before or with a recurrence of disease (Buffé *et al.*, 1970).

*$\beta$S-Foetoprotein*

Takahashi and his colleagues (1967) described $\beta$S-foetoprotein which occurs in elevated amounts in the serum of patients with hepatocarcinoma (10/23), cholangiocarcinoma (1/5), gastric carcinoma (2/7) and leukaemia and lymphoma (2/7) (Wada *et al.*, 1971). As with the other foetal antigens, raised serum values can also be detected in liver diseases such as cirrhosis.

Double diffusion agar methods and immunoelectrophoresis have been used to demonstrate this foetoprotein which occurs in foetal serum and tissues, including the liver, disappearing from the body fluids by the fifth–seventh post-natal month. Preliminary studies have shown that it is a glycoprotein whose molecular weight probably exceeds 200,000. In contrast to CEA, it is located in the cytoplasm of foetal hepatocytes. The behaviour of this material in response to anti-tumour therapy has not been reported.

*Leukaemia-associated antigens (LAA)*

Within embryonic tissues and serum are macromolecules that are also present in the serum of approximately one-third of patients with various types of leukaemia (Harris *et al.*, 1971). These leukaemia-associated antigens (LAA) seem to be derived from the tumour cell membrane and are also present in the sera of some patients with Hodgkin's disease. Using double diffusion and cross-over electrophoretic methods, LAA are not seen in the sera of normal persons or of patients with hepatomata. Unfortunately, serum LAA levels need not decline during remission. Further studies are required to outline the value of these materials in the clinical management of leukaemia.

*Heterophile foetal antigen*

Edynak and his associates (1970) have reported a heterophile antigen in foetal but not neonatal or adult sera, except for the sera of 17 of 200 patients with various tumours, including leukaemia. This material is capable of eliciting an antibody response in about 1% of patients with various types of cancer and this antibody can precipitate saline extracts of many diverse types of human tumours, including those arising from breast, colon, ovary, kidney, muscle and bone.

While the antigen does not occur in the serum of healthy persons, it can be detected in some simple tumours and some non-neoplastic tissues. It has the electrophoretic mobility of a $\gamma$-globulin and it has been suggested that it and the LAA may be related (Alexander, 1972). To date, the effects of therapy upon its levels are unknown; if more sensitive techniques do not become available, its low incidence in neoplasia makes it unlikely to have clinical usage.

*Foetal sulphoglycoprotein antigen (FSA)*

Three sulphoglycoproteins are known to appear in the human foetal gastro-intestinal tract about the seventh or eighth week of intrauterine development (Häkkinen, Korhonen and Saxen, 1968). One of them, located in the cells of the superficial epithelium of the foetal stomach, disappears from this site 9 months after birth and is called foetal sulphoglycoprotein antigen (FSA).

Häkkinen and Viikari (1969) reported that 96% of patients with gastric carcinomata contain FSA in the gastric juice. Immunological studies showed that FSA was present in the tumour cells and in the superficial cells of the mucosa in relation to the tumours (Häkkinen, Jarvi and Gironroos, 1968). It was also found, using double diffusion methods at this site, in 14% of subjects with peptic ulceration and in some patients with non-epithelial neoplasms. FSA production was also noted to apparently precede the development of overt gastric carcinoma. While this is a most interesting and important observation, the incidence of FSA-positive patients with peptic ulceration far exceeds their known incidence of malignant change of approximately 2%. Further chemical and immunological studies of this material and its relationship to intestinal metaplasia are needed. Unfortunately, successful removal of a gastric tumour need not be followed by a decline of FSA levels in gastric juice.

*Carcinoplacental alkaline phosphatase*

The induction of experimental neoplasia is frequently associated with the activation of isozymes characteristic of foetal or neonatal organs. Generally, there is a quantitative change toward the foetal pattern, *e.g.* lactate dehydrogenase, aldolase (Leese, 1969). While these observations have important basic connotations in the field of differentiation, of great clinical importance are qualitative isozymic alterations which are also reflected in the body fluids. To date, the only isozyme fitting the latter category is the placental alkaline phosphatase.

At least 5 organ-specific isozymes of alkaline phosphatase are known to occur in the serum and are derived from the liver, bones, lung, intestinal tract and placenta. The placental isozyme does not occur in foetal tissues or serum but is present in maternal serum during the third trimester of pregnancy. It is characterized by being heat stable and inhibited by L-phenylalanine, in contrast to the other forms (Fishman *et al.*, 1968), and is never found in the serum of normal *male* subjects.

The isozyme, first detected in a patient named Regan with bronchial carcinoma, occurs in the serum of a minority of patients with a wide variety of neoplasms (Table VIII). In addition, many of the important pre-cancerous disorders or diseases which feature in the differential diagnosis of neoplasia can result in " false positive " results (Table VIII).

TABLE VIII.—*Incidence of Regan Isozyme of Alkaline Phosphatase in the Sera of Patients with a Variety of Disorders*†

| Disorder | Incidence of Regan isozyme |
|---|---|
| Bronchial carcinoma | 7/51 |
| Mammary carcinoma | 6/49 |
| Genito-urinary carcinoma | 10/55 |
| Lymphoma | 2/25 |
| Malignant melanoma | 0/5 |
| Gastro-intestinal carcinoma | 10/81 |
| Alcoholic cirrhosis* | 2/8 |
| Hepatitis* | 0/6 |
| Ulcerative colitis* | 2/4 |
| Diverticulitis* | 1/1 |
| Hydronephrosis* | 1/2 |

* From Nathanson and Fishman, 1971.

† These patients form a selected rather than a random group of controls as each was found to have a raised serum alkaline phosphatase level by routine testing. The presence or absence of the Regan isozyme was then sought.

This tumour-produced isozyme has the same immunological, electrophoretic and biochemical properties as the placental isozyme (Nathanson and Fishman, 1971) and has also been shown to be a product of Hela cells (Griffin, Cox and Grujic, 1967).

When present, this isozyme is clinically useful to monitor tumour progression or regression with therapy and can also be detected in malignant serosal exudates. It would seem that all patients with unexplained raised serum alkaline phosphatase levels determined by the routine methods should have the serum reexamined to determine if the placental isozyme is responsible for the aberrations. A positive result would necessitate detailed clinical examination to detect or exclude a latent neoplasm.

A variant of this carcinoplacental alkaline phosphatase isozyme has been detected by Warnock and Reesman (1969). It occurs in the serum of some patients with hepatoma but not cholangiocarcinoma and/or liver damage, but only when AFP levels are also raised (Portugal, Azevedo and Manso, 1970). This isozyme is different from the typical liver alkaline phosphatase and is not produced by the foetal liver.

*Hormones*

The ectopic hormone syndromes represent a vast and important aspect of oncology which is outside the scope of this present article. They have been recognized with increasing frequency in recent years, and Ellison and Neville (1972) consider ectopic hormone production to be a disorder due to non-random change in gene expression, and believe that this mechanism also accounts for foetal antigen production. This thesis is given added credence when it is appreciated that non-trophoblast containing tumours can form substances usually of placental origin. These include gonadotrophins (Castleman, Scully and McNeely, 1972), human placental lactogen (HPL) (Weintraub and Rosen, 1971) and plasminogen activators (Davidson *et al.*, 1969).

Bronchial tumours are most frequently implicated but HPL has also been noted to be a product of hepatoma and hepatoblastoma (Weintraub and Rosen, 1971), both of which also can produce AFP (Table V).

Inappropriate hormone production also serves as an index of tumour activity and the levels decline with successful therapy. It would seem worthwhile to consider including a battery of hormonal immunoassays in all assessments of the functional activity of tumours.

*Foetal macromolecules present only in tumours*

Many different human tumours have been found to contain foetal antigens, which to date have not been shown to pass from the tumours into the body fluids.

Klavins and his colleagues (1971) have declared that immunologically definable embryonic cell components are present in all human carcinomata. In favour of this concept, which has support in the field of experimental neoplasia (Stonehill and Bendich, 1970), they were able to demonstrate that absorbed anti-sera raised against whole human 6–7 week old embryos cross-reacted with extracts of lung, breast, colonic, hepatic, renal, bronchial and skin carcinomata. No reactions were observed with normal tissues except the epidermis. In keeping with the hypothesis that these manifestations are examples of retrogenetic expression, it has been noted that the foetal and tumour materials exhibit immunological identity and have similar gel filtration properties giving a molecular weight of the order of 66–68,000 (Klavins *et al.*, 1971).

Two large macromolecular complexes, one of which is also present in foetal tissue, have been isolated by Yashi *et al.* (1968) from bronchial, gastric, pancreatic, renal and hepatic carcinomata. Other gastric and colonic carcinomata were shown to have different but related antigens. These molecules, despite diligent search, do not occur in the body fluids. They are insoluble in perchloric acid, although they show slight staining with periodic acid–Schiff's reagents, and are heat labile and alcohol insoluble.

The presence of antigens common to foetal tissues and tumours, including those of colonic, mammary and pancreatic origin, has also been recorded by Tee and his colleagues (Tee *et al.*, 1965; Barnes and Tee, 1971). In addition, 2 non-foetal antigens were demonstrated by them in those tumours. Trouillas (1971) found that glioblastoma and astrocytoma contain foetal brain macromolecules which are soluble in saline and appear to contain lipid.

The discovery of each of these macromolecules, although of no immediate practical clinical application, is of great interest and importance. However, it seems to us essential that their relationship to one another and to the other ones previously described should be ascertained in the near future. Moreover, their chemical nature and site in or on tumour cells require clarification.

### PROSPECTS

In this review, we have been discussing those presently known foetal antigens which occur in association with human neoplasia. Almost certainly the next few years will prove that the list is far from complete. It seems imperative to us that if confusion of terminology and duplication of research effort is to be avoided, a co-ordinated scheme for distributing material to interested workers should be established within the near future. In this way, new materials will be assessed quickly, their relationship to others outlined and their place in clinical practice examined in an integrated and detailed manner.

Another collaborative project would be to define the specificities of the antibodies used in reactions to detect and evaluate the foetal antigens. The gel diffusion method contains certain inbuilt safeguards against the possibility that more than one antigen will be included in the evaluation of a given case. With radioimmunoassay, it may be difficult to distinguish between a highly active antigen present in nanogram amounts and a second cross-reacting antigen with a concentration a thousand times greater.

A third study might consider whether the concept of purity as normally understood with chemical products is meaningful in the field of carbohydrate-tumour antigens. It is known, for example, that tumour surface antigens differ from the normal in part by their distribution and availability, as defined by steric properties of the cell surface. A similar steric relationship may occur at a molecular level in determining accessibility of groupings on the antigen to antibody molecules.

It will be of interest to know if these antigens have any role to play in indicating tumour aetiology. Human tumours, like animal tumours, seem to possess antigens peculiar to one tumour or common to different tumours of similar or differing histology and at various sites. The evidence from experimental animal studies that shared antigens may be indicative of similar aetiology, such as a virus, has profound meaning for human neoplasia.

The field of histopathology may benefit from study of foetal antigens and related substances. At present, most tumours are classified according to their histogenesis and behaviour, functional attributes, with the exception of endocrine tumours, seldom being included. The finding of new macromolecules, with or without metabolic activity in or on tumour cells, may facilitate the development of a new era of functional pathology. By outlining the immunological spectra, functional heterogeneity between tumours of identical light morphology or between different cells of the same tumour may be discerned, which could have aetiological behavioural and prognostic significance.

It would seem to be worth while ascertaining if the prognosis of CEA-positive and CEA-negative tumours is different. Recent experimental work has related the metastasizing capacity of tumours to a loss of surface glycoprotein with the plasma (Kim and Carruthers, 1972). By following up patients after surgery for malignant disease, it should be feasible to discover if those tumours associated with raised plasma levels tend to metastasize earlier than those with normal levels. This seems to be true for teratomata and AFP production (Mawas *et al.*, 1971). The precise histogenesis of some tumours remains to be ascertained and knowledge of the embryonic sites of foetal antigen production may help with such problems. As an example, it is not unreasonable to propose that the so-called endodermal sinus tumour, if it is of vitelline origin (Teilum, 1971), should produce AFP.

The immunological diagnosis of neoplasia will probably become one of the major research-orientated aspects of clinical pathology in the next decade. At the beginning of this review, we proposed that foetal antigens would require to fulfil 4 criteria to be of clinical value. Unfortunately, none yet satisfy them all, failing at least in regard to tumour specificity. Nonetheless, materials like CEA and AFP have a clinical role at this point in time, aiding with diagnosis (especially AFP) and monitoring the effects of therapy. It still remains, however, to be ascertained if longevity and morbidity will be improved by detecting and treating recurrent disease earlier than usual.

Most tumours are considered curable if treated early by surgery, radiotherapy and/or chemotherapy and hence many clinical scientists are searching for a screening test for early cancer. Whether or not any single material will be found to fulfil this role is uncertain. Such an approach is more likely to be fruitful if a series of different tumour products are measured. A continuing search for CEA-like materials in other human tumours seems warranted. Our own studies (at the Institute of Cancer Research) have revealed the presence in some gastro-intestinal, mammary and bronchial tumours and the corresponding sera of another macromolecule which is absent from, or present only in trace amounts in, control tissues. This material (called " X ") is soluble in perchloric acid, and occurs either alone or in conjunction with CEA from which it has been separated. We are attempting to prepare appropriate antisera and establish an immunoassay. By developing radioimmunoassays for each new tumour product, if preliminary studies are encouraging, and using them in conjunction with those including hormones or hormonal fragments which are already established, a battery of tests will become available which, taken together, may aid in detection, differential diagnosis and prognostication.

### REFERENCES

Abelev, G. I. (1968) Production of Embryonal Serum α-globulins by Hepatomas: Review of Experimental and Clinical Data. *Cancer Res.*, **28**, 1344.

Abelev, G. I. (1971) Alpha-foetoprotein in Ontogenesis and Its Association with Malignant Tumours. *Adv. Cancer Res.*, **14**, 295.

Abelev, G. I. & Bakirov, R. (1967) *In vitro* Synthesis of Serum Embryonic Antigens by the Liver. *Vop. med. Khim.*, **13**, 378.

Abelev, G. I., Perova, S. D., Khramkova, N. I., Postnikova, Z. A. & Irlin, I. S. (1963) Production of Embryonal α-globulin by Transplantable Mouse Hepatomas. *Transplantation*, **1**, 174.

Abelev, G. I., Perova, S. D., Bakirov, R. D. & Irlin, I. S. (1967) Further Study of Embryonic Serum α-globulin Synthesised by Hepatomas. In *Specific Tumour Antigens*, Vol. 2. Ed. R. J. G. Harris. UICC Monograph Series. Copenhagen: Munksgaard. p. 32.

Abelev, G. I., Tsvetkov, V. S., Biryulina, T. I., Elgort, D. A., Olovnikov, A. M., Gusev, A. I., Yazova, A. K., Perova, S. D., Rubtsov, I. V., Kantorovich, B. A., Tur, V., Khasanov, A. I. & Levina, D. M. (1971) Evaluation of the Use of Highly Sensitive Methods for Determination of α-foetoprotein in the Diagnosis of Hepatocellular Cancer and Teratoblastoma. *Byull. eksp. Biol. Med.*, **71**, 75.

Alexander, P. (1972) Foetal "Antigens" in Cancer. *Nature, Lond.*, **235**, 137.

Alpert, E., Pinn, V. W. & Isselbacher, K. J. (1971) Alpha-foetoprotein in a Patient with Gastric Carcinoma Metastatic to the Liver. *New Engl. J. Med.*, **285**, 1058.

Bakirov, R. D. (1968) Appearance of Embryonic Serum Alpha-globulin in Adult Mice after Carbon Tetrachloride Poisoning. *Byull. eksp. Biol. Med.*, **65**, 45.

Baldwin, R. W. (1970) Tumour Specific Antigens Associated with Chemically Induced Tumours. *Rev. Eur. Études clin. biol.*, **25**, 593.

Barnes, S. J. & Tee, D. E. H. (1971) The Occurrence of Certain Carcinoembryonic Antigens in Man. In *Protides of the Biological Fluids*. Ed. H. Peeters. Oxford: Pergamon Press. p. 281.

Bennhold, H. (1962) Überblick Über die Transportfunktion der Serumeisweisskörper. In *Protides of the Biological Fluids*. Ed. H. Peeters. Amsterdam: Elsevier Publ. Corp. p. 58.

Bennhold, H. & Kallee, E. (1959) Comparative Studies on the Half-life of $I^{131}$-labeled Albumin and Non-radioactive Human Serum Albumin in a Case of Analbuminaemia. *J. clin. Invest.*, **58**, 863.

Bergmann, F. M., Levine, L. & Spiro, R. G. (1962) Fetuin: Immuno Chemistry and Quantitative Estimation in Serum. *Biochim. Biophys. Acta*, **58**, 41.

Brawn, R. J. (1970) Possible Association of Embryonal Antigen(s) with Several Primary 3-methylcholanthrene-induced Murine Sarcomas. *Int. J. Cancer*, **6**, 245.

Broberger, O. & Perlmann, P. (1959) Autoantibodies in Human Ulcerative Colitis. *J. exp. Med.*, **110**, 657.

Bubenik, J., Perlmann, P., Helmstein, K. & Moberger, G. (1970) Immune Response to Urinary Bladder Tumors in Man. *Int. J. Cancer*, **5**, 39.

Buffé, D., Rimbault, C., Lemerle, J., Schweisguth, O. & Burtin, P. (1970) Presence d'une Ferroproteine d'origine Tissulaire, L'$\alpha_2$H, dans le Serum des Enfants Porteurs de Tumeurs. *Int. J. Cancer*, **5**, 85.

Burtin, P., von Kleist, S. & Sabine, C. (1971) Loss of Normal Colonic Membrane Antigen in Human Cancers of the Colon. *Cancer Res.*, **31**, 1038.

Burtin, P., Martin, E., Sabine, M. C. & von Kleist, S. (1972) Immunological Study of Polyps of the Colon. *J. natn. Cancer Inst.*, **48**, 25.

Castleman, B., Scully, R. E. & McNeely, B. W. (1972) Case Records of Massachusetts General Hospital. Case 13–1972. *New Engl. J. Med.*, **286**, 713.

Coggin, J. H., Ambrose, K. R. & Anderson, N. G. (1970) Fetal Antigen Capable of Inducing Transplantation Immunity Against SV40 Hamster Tumour Cells. *J. Immun.*, **105**, 524.

Coligan, J. E., Lautenschleger, J. T., Egan, M. L. & Todd, C. W. (1972) Isolation and Characterisation of Carcinoembryonic Antigen. *Immunochemistry*, **9**, 377.

Collatz, E., von Kleist, S. & Burtin, P. (1971) Further Investigation of Circulating Antibodies in Colon Cancer Patients: on the Autoantigenicity of the Carcinoembryonic Antigen. *Int. J. Cancer*, **8**, 298.

Crichlow, R. W. & White, R. R. (1970) Search for Carcinoembryonic Antigen (CEA) in Adenomas of the Colon. *Proc. Am. Ass. Cancer Res.*, **11**, 18.

Davidson, J. F., McNicol, G. P., Frank, G. L., Anderson, T. J. & Douglas, A. S. (1969) Plasminogen-activator-producing Tumour. *Br. med. J.*, i, 88.

Duff, R. & Rapp, F. (1970) Reaction of Serum from Pregnant Hamsters with Surface of Cells Transformed by SV40. *J. Immun.*, **105**, 521.

Edynak, E. M., Hirshaut, Y., Old, L. J. & Trempe, G. L. (1971) Antigens of Human Breast Cancer. *Proc. Am. Ass. Cancer Res.*, **12**, 75.

Edynak, E. M., Old, L. J., Vrana, M. & Lardis, M. (1970) A Fetal Antigen in Human Tumours Detected by an Antibody in the Serum of Cancer Patients. *Proc. Am. Ass. Cancer Res.*, **11**, 22.

EGAN, M. L., LAUTENSCHLEGER, J. T., COLIGAN, J. E. & TODD, C. W. (1972) Radioimmune Assay of Carcinoembryonic Antigen. *Immunochemistry*, **9**, 289.

ELLISON, M. L. & NEVILLE, A. M. (1972) Neoplasia and Ectopic Hormone Production. In *Modern Trends in Oncology*. Ed. R. Raven. London: Butterworths (in press).

ENGELHARDT, N. V., SHIPOVA, L. YA., GUSEV, A. I., YAZOVA, A. K. & TER-GRIGOROVA, E. N. (1969) Observations on the $\alpha_F$-globulin in Sections of Human Embryo Liver and the Liver of Newborn Mice by the Fluorescent Antibody Method. *Byull. eksp. Biol. Med.*, **68**, 62.

FISHMAN, W. H., INGLIS, N. R., GREEN, S., ANTISS, C. L., GOSH, N. K., REIF, A. E., RUSTIGAN, R., KRAUT, M. J. & STOLBACH, L. L. (1968) Immunology and Biochemistry of Regan Isoenzyme of Alkaline Phosphatase in Human Cancer. *Nature, Lond.*, **219**, 697.

FREED, D. L. J. & TAYLOR, G. (1972) Carcinoembryonic Antigen in Faeces. *Br. med. J.*, i, 85.

GASIC, G. & GASIC, T. (1962) Removal and Regeneration of the Cell Coating in Tumour Cells. *Nature, Lond.*, **196**, 170.

GIBBS, N. M. & FRENCH, L. W. (1971). The Colon Mucus Antibody. *J. clin. Path.*, **24**, 867.

GITLIN, D. (1971) Sites of Alpha-foetoprotein Synthesis. *New Engl. J. Med.*, **285**, 1436.

GITLIN, D. & BOESMAN, M. (1966) Serum Alpha-foetoprotein, Albumin and γG-globulin in the Human Conceptus. *J. clin. Invest.*, **45**, 1826.

GITLIN, D. & BOESMAN, M. (1967*a*) Fetus Specific Proteins in Several Mammals and Their Relation to Human α-Fetoprotein. *Comp. Biochem. Physiol.*, **21**, 327.

GITLIN, D. & BOESMAN, M. (1967*b*) Sites of Serum Alpha-fetoprotein Synthesis in the Human and in the Rat. *J. clin. Invest.*, **46**, 1010.

GITLIN, D. & PERICELLI, A. (1970) Synthesis of Serum Albumin, Prealbumin, α-Fetoprotein, $\alpha_1$-Antitrypsin and Transferrin by the Human Yolk Sac. *Nature, Lond.*, **228**, 995.

GOLD, P., (1967) Circulating Antibodies against Carcinoembryonic Antigens of the Human Digestive System. *Cancer, N.Y.*, **20**, 1663.

GOLD, P. (1971) Antigenic Reversion in Human Cancer. *A. Rev. Med.*, **22**, 85.

GOLD, P. & FREEDMAN, S. O. (1965*a*) Demonstration of Tumor-specific Antigens in Human Colonic Carcinoma by Immunological Tolerance and Absorption Technique. *J. exp. Med.*, **121**, 439.

GOLD, P. & FREEDMAN, S. O. (1965*b*) Specific Carcinoembryonic Antigens of the Human Digestive System. *J. exp. Med.*, **122**, 467.

GOLD, P., GOLD, M. & FREEDMAN, S. O. (1968) Cellular Location of Carcinoembryonic Antigens of the Human Digestive System. *Cancer Res.*, **28**, 1331.

GOLD, P., KRUPEY, J. & ANSARI, H. (1970) Position of the Carcinoembryonic Antigen of the Human Digestive System in Ultrastructure of Tumor Cell Surface. *J. natn. Cancer Inst.*, **45**, 219.

GRIFFIN, M. J., COX, R. P. & GRUJIC, N. (1967) A Chemical Method for the Isolation of Hela Cell Nuclei and the Nuclear Localisation of Hela Cell Alkaline Phosphatase. *J. Cell Biol.*, **33**, 200.

GUSEV, A. I., ENGELHARDT, N. V., MASSEYEFF, R., CAMAIN, R. & BASTERIS, B. (1971) Immunofluorescent Study of Alpha-foetoprotein (αfp) in Liver and Liver Tumours. II. Localisation of αfp in the Tissues of Patients with Primary Liver Cancer. *Int. J. Cancer*, **7**, 207.

HAKOMORI, S. & MURAKAMI, W. T. (1968) Glycolipids of Hamster Fibroblasts and Derived Malignant Transformed Cell Lines. *Proc. natn. Acad. Sci. U.S.A.*, **59**, 254.

HÄKKINEN, I., JARVI, O. & GIRONROOS, J. (1968) Sulphoglycoprotein Antigens in the Human Alimentary Canal and Gastric Cancer. An Immunohistochemical Study. *Int. J. Cancer*, **3**, 572.

HÄKKINEN, I., KORHONEN, L. K. & SAXEN, L. (1968) The Time of Appearance and Distribution of Sulphoglycoprotein Antigens in the Human Foetal Alimentary Canal. *Int. J. Cancer*, **3**, 582.

HÄKKINEN, I. & VIIKARI, S. (1969) Occurrences of Fetal Sulphoglycoprotein Antigens in the Gastric Juice of Patients with Gastric Diseases. *Ann. Surg.*, **169**, 277.

HALL, R. R., LAURENCE, D. J. R., DARCY, D., STEVENS, U., JAMES, R., ROBERTS, S. & NEVILLE, A. M. (1972) Carcinoembryonic Antigen (CEA) in the Urine of Patients with Urothelial Carcinoma. *Br. med J.* (in the press).

HARRIS, R., VIZA, D., TODD, R., PHILLIPS, J., SUGAR, R., JENNISON, R. F., MARRIOTT, G. & GLEESON, M. H. (1971) Detection of Human Leukaemia Associated Antigens in Leukaemic Serum and Normal Embryos. *Nature, Lond.*, **233**, 556.

HAVERBACK, B. J. & DYCE, B. J. (1972) *Presence of CEA(s) in Bronchogenic Carcinoma.* Second Fetal Antigen Conference. U.S. Atomic Energy Commission Report. Ed. N. G. Anderson and J. H. Coggin (in press).

HELLSTRÖM, I., HELLSTRÖM, K. E., PIERCE, G. E. & BILL, A. H. (1968) Demonstration of Cell-bound and Tumoral Immunity Against Neuroblastoma Cells. *Proc. natn. Acad. Sci. U.S.A.*, **60**, 1231.

HIRSZFELD, L. (1929) Untersuchungen über die serologischen Eigenschaften der Gewebe: über serologische Eigenschaften der Neubildungen. *Z. ImmunForsch. exp. Ther.*, **64**, 81.

HOUSTEK, J., MASOPUST, J., KITHIER, K. & RADL, J. (1968) Hepatocellular Carcinoma in Association with a Specific Fetal Alpha-1-globulin Fetoprotein. *J. Pediat.*, **72**, 186.

HULL, E., CARBONE, P., GITLIN, D., O'GARA, R. & KELLY, M. (1969) Alpha-fetoprotein in Monkeys with Hepatoma. *J. natn. Cancer Inst.*, **42**, 1035.

JEHN, U. W., NATHANSON, L., SCHWARTZ, R. S. & SKUMEN, M. (1970) *In vitro* Lymphocyte Stimulation by a Soluble Antigen from Malignant Melanoma. *New Engl. J. Med.*, **283**, 329.

KARITZKY, D. & BURTIN, P. (1967) Isolement de l'autoantigene, Responsable de la Formation d'autoanticorps chez les Malades Atteints de Cancer Gastrique. *Eur. J. Biochem.*, **1**, 411.

KHASANOV, A. J., ABELEV, G. I., PEROVA, S. D., POLENKO, V. K., RYAPSOVA, U. K. & SHIRENKOVA, O. (1971) quoted by Abelev, G. I. (1971).

KIM, U. & CARRUTHERS, C. (1972) Relationship between Glycocalyx Dissociability Surface Antigen Solubility, Immunogeneity and Metastasising Capacity of Mammary Carcinoma. *Proc. Am. Ass. Cancer Res.*, **13**, 69.

KIRSH, I., WISE, R. & OLIVER, I. (1967) Postalbumin —a Fetal Specific Rat Plasma Protein. *Biochem. J.*, **102**, 763.

KITHIER, K., MASOPUST, J. & RADL, J. (1968) Fetal α-globulin of Bovine Serum Differing from Fetuin. *Biochim. Biophys. Acta*, **160**, 135.

KLAVINS, J. V., MESA-TEJADA, R. & WEISS, M. (1971) Human Carcinoma Antigens Cross-reacting with Anti-embryonic Antibodies. *Nature, Lond.* (*New Biology*), **234**, 153.

KLEIN, G., CLIFFORD, P., HENLE, G., HENLE, W., GEERING, G. & OLD, L. J. (1969) EBV-associated Serological Patterns in a Burkitt Lymphoma Patient during Regression and Recurrence. *Int. J. Cancer*, **4**, 416.

KLEINMAN, M. S., HARWELL, L. & TURNER, M. D. (1971) Studies of Colonic Carcinoma Antigens. *Gut*, **12**, 1.

VON KLEIST, S., BUFFÉ, D. & BURTIN, P. (1968) Relation entre les Antigenes du Liquide Amniotique et du Serum Foetal. *Clin. chim. Acta*, **20**, 89.

VON KLEIST, S. & BURTIN, P. (1966) On the Specificity of Autoantibodies Present in Colon Cancer Patients. *Immunology*, **10**, 507.

VON KLEIST, S. & BURTIN, P. (1969*a*) Isolation of a Fetal Antigen from Human Colonic Tumors. *Cancer Res.*, **29**, 1961.

VON KLEIST, S. & BURTIN, P. (1969*b*) Localisation Cellulaire d'un Antigene Embryonnaire de Tumeurs Coliques Humaines. *Int. J. Cancer*, **4**, 874.

KOZOWER, M., FAWAZ, K. A., MILLER, H. M. & KAPLAN, M. M. (1971) Positive Alpha-fetoglobulin in a Case of Gastric Carcinoma. *New Engl. J. Med.*, **285**, 1059.

KRAEMER, P. M. (1966) Regeneration of Sialic Acid on the Surface of Chinese Hamster Cells in Culture. I. General Characteristics of the Replacement Process. *J. cell comp. Physiol.*, **68**, 85.

KROES, R. & WEISBURGER, J. H. (1972) Precocious Appearance of α-fetal Protein in Serum of Rats given Hepatocarcinogens. *Am. Cancer Soc. Abstracts*, **13**, 53.

KRUPEY, J., GOLD, P. & FREEDMAN, S. O. (1967) Purification and Characterisation of Carcinoembryonic Antigens of the Human Digestive System. *Nature, Lond.*, **215**, 67.

KRUPEY, J., GOLD, P. & FREEDMAN, S. O. (1968) Physiochemical Studies of the Carcinoembryonic Antigen of the Human Digestive System. *J. exp. Med.*, **128**, 387.

LAURENCE, D. J. R., STEVENS, U., BETTELHEIM, R., DARCY, D., LEESE, C., TURBERVILLE, C., ALEXANDER, P., JOHNS, E. W. & NEVILLE, A. M. (1972) Evaluation of the Role of Plasma Carcinoembryonic Antigen (CEA) in the Diagnosis of Gastrointestinal, Mammary and Bronchial Carcinoma. *Br. med. J.* (in the press).

LEESE, C. L. (1969) Enzymes and Isoenzymes. *Ciba Foundation Symposium on Homoeostatic Regulators*. Ed. G. E. W. Wolstenholme & J. Knight. p. 144.

LEVI, M. M., KELLER, S. & MANDL, I. (1969) Antigenicity of a Papillary Serous Cystadenocarcinoma Tissue Homogenate and its Fractions. *Am. J. Obstet. Gynec.*, **105**, 856.

LOGERFO, P., HERTER, F. P. & BENNETT, S. J. (1972*a*) Absence of Circulating Antibodies to Carcinoembryonic Antigens in Patients with Gastro-intestinal Malignancies. *Int. J. Cancer*, **9**, 344.

LOGERFO, P., KRUPEY, J. & HANSEN, H. J. (1971) Demonstration of an Antigen Common to Several Varieties of Neoplasia. *New Engl. J. Med.*, **285**, 138.

LOGERFO, P., LOGERFO, F., HERTER, F., BARKER, H. G. & HANSEN, H. J. (1972*b*) Tumor-associated Antigens in Patients with Carcinoma of the Colon. *Am. J. Surg.*, **123**, 127.

MCNEIL, C., LADLE, J. N., HELMICK, W. M., TRENTELMAN, E. & WENTZ, M. W. (1969) An Antiserum to Ovarian Mucinous Cyst Fluid with Colon Cancer Specificity. *Cancer Res.*, **29**, 1535.

MARTIN, J. P., CHARLIONET, R. & ROPARTZ, C. (1971) The Presence of $\alpha_2$H in Sera from Patients with Malignant Haemopathies and Cirrhosis. *Rev. Eur. Études clin. biol.*, **26**, 266.

MARTIN, F. & MARTIN, M. S. (1970) Demonstration of Antigens Related to Colonic Cancer in the Human Digestive System. *Int. J. Cancer*, **6**, 352.

MASOPUST, J., KITHIER, K., RADL, J., KOUTECKY, J. & KOTAL, L. (1968) Occurrence of Alpha-foetoprotein in Patients with Neoplasms and Non-neoplastic Diseases. *Int. J. Cancer*, **3**, 364.

MASOPUST, J., ZIZKOVSKY, V. & KITHIER, K. (1971) Fetoproteins in Different Animal Species. In *Protides of the Biological Fluids*. Ed. H. Peeters. Oxford: Pergamon Press. p. 63.

MAWAS, C., BUFFÉ, D., SCHWEISGUTH, O. & BURTIN, P. (1971) Quoted by Abelev, G. I. (1971).

MEHLMAN, D. J., BULKLEY, B. H. & WIERNIK, P. H. (1971) Serum Alpha$_1$-fetoglobulin with Gastric and Prostatic Carcinoma. *New Engl. J. Med.*, **285**, 1060.

MOORE, T. L., KUPCHIK, H. Z., MARCON, N. & ZAMCHEK, N. (1971) Carcinoembryonic Antigen Assay in Cancer of the Colon and Pancreas and Other Digestive Tract Disorders. *Am. J. dig. Dis.*, **16**, 1.

MORTON, D. L., MALMGREN, R. A., HALL, W. T. & SCHIDLOVSKY, G. (1969) Immunologic and Virus Studies with Human Sarcomas. *Surgery, St Louis*, **66**, 152.

MORTON, D. L., MALMGREN, R. A., HOLMES, E. C. & KETCHAM, A. S. (1968) Demonstration of Antibodies Against Human Malignant Melanoma by Immunofluorescence. *Surgery, St Louis*, **64**, 233.

NAIRN, R. C., FOTHERGILL, J. E., MCENTEGART, M. G. & RICHMOND, H. G. (1962) Loss of Gastro-intestinal-specific Antigen in Neoplasia. *Br. med. J.*, i, 1791.

NATHANSON, L. & FISHMAN, W. H. (1971) New Observations on the Regan Isoenzyme of Alkaline Phosphatase in Cancer Patients. *Cancer, N.Y.*, **27**, 1388.

NISHI, S. (1970) Isolation and Characterisation of a Human Fetal α-globulin from the Sera of Fetuses and a Hepatoma-patient. *Cancer Res.*, **30**, 2507.

NISHIOKA, M., IBATA, T., OKITA, K., HARADA, T. & FUJITA, T. (1972) Localisation of α-Fetoprotein in Hepatoma Tissues by Immunofluorescence. *Cancer Res.*, **32**, 162.

O'CONOR, G. T., TATARINOV, Y. S., ABELEV, G. I. & URIEL, J. (1970) A Collaborative Study for the Evaluation of a Serological Test for Primary Liver Cancer. *Cancer, N.Y.*, **25**, 1091.

OLD, L. J. & BOYSE, E. A. (1964) Immunology of Experimental Tumors. *A. Rev. Med.*, **15**, 167.

ORDER, S. E., PORTER, M. & HELLMAN, S. (1971) Hodgkin's Disease: Evidence for a Tumor-associated Antigen. *New Engl. J. Med.*, **285**, 471.

PANTELOURIS, E. M. & HALE, P. A. (1962) Developmental Changes in the Plasma Protein Pattern of the Mouse. *Nature, Lond.*, **195**, 79.

PEARSON, G. & FREEMAN, G. (1968) Evidence Suggesting a Relationship Between Polyma Virus-induced Transplantation Antigen and Normal Embryonic Antigen. *Cancer Res.*, **28**, 1665.

PEROVA, S. D., ELGORT, D. A. & ABELEV, G. I. (1971) Alpha-fetoprotein in the Serum of the Rat after Partial Hepatectomy. *Byull. eksp. Biol. Med.*, **71**, 45.

PORTUGAL, M. L., AZEVEDO, M. S. & MANSO, C. (1970) Serum Alpha-fetoprotein and Variant Alkaline Phosphatase in Human Hepatocellular Carcinoma. *Int. J. Cancer*, **6**, 383.

PURVES, L. R., BERSOHN, I. & GEDDES, E. W. (1970*b*) Serum Alpha-fetoprotein and Primary Cancer of the Liver in Man. *Cancer*, **25**, 1261.

PURVES, L. R., MACNAB, M. & BERSOHN, I. (1968) Serum Alpha-fetoprotein. I. Immunodiffusion and Immunoassay Results in Cases of Primary Cancer of the Liver. *S. Afr. J. Med.*, **42**, 1138.

PURVES, L. R., VAN DER MERWE, E. & BERSOHN, I. (1970*a*) Serum Alpha-fetoprotein. V. The Bulk Preparation and Some Properties of α-Fetoprotein Obtained from Patients with Primary Cancer of the Liver. *S. Afr. J. Med.*, **44**, 1264.

REYNOSO, G., CHU, T. M., HOLYOKE, D., COHEN, E., NEMOTO, T., WANG, J.-J., CHUANG, J., GUINAN, P. & MURPHY, G. (1972) Carcinoembryonic Antigen in Patients with Different Cancers. *J. Am. med. Ass.*, **220**, 361.

RUOSLAHTI, E. & SEPPÄLÄ, M. (1971) Studies of Carcino-fetal Proteins. III. Development of a Radioimmunoassay for α-Fetoprotein. Demonstration of α-Fetoproteins in Serum of Healthy Human Adults. *Int. J. Cancer*, **8**, 374.

RUOSLAHTI, E., SEPPÄLÄ, M., PIHKO, H. & VUOPIO, P. (1971) Studies of Carcinofetal Proteins. II. Biochemical Comparison of α-Fetoprotein from Human Fetuses and Patients with Hepatocellular Cancer. *Int. J. Cancer*, **8**, 283.

SEPPÄLÄ, M. & RUOSLAHTI, E. (1972*a*) Radioimmunoassay of Maternal Serum Alphafetoprotein During Pregnancy and Delivery. *Am. J. Obstet. Gynec.*, **112**, 208.

SEPPÄLÄ, M. & RUOSLAHTI, E. (1972*b*) α-Fetoprotein in Normal and Pregnancy Sera. *Lancet*, i, 375.

SMITH, R. T., KLEIN, G. & KLEIN, E. (1967) Studies of the Membrane Phenomenon in Cultured and Biopsy Cell Lines from the Burkitt Lymphoma. In *Advances in Transplantation. Ed.* J. Dausset, J. Hamberger & G. Mathe. Baltimore: Williams and Wilkins. p. 483.

SPIRO, R. G. (1960) Studies on Fetuin, a Glycoprotein of Fetal Serum. I. Isolation, Chemical Composition and Physicochemical Properties. *J. biol. Chem.*, **235**, 2860.

SPIRO, R. G. (1970) Glycoproteins. *A. Rev. Biochem.*, **39**, 599.

STANISLAWSKI–BIRENCWAJG, M. (1967) Specific Antigens of Rat Embryonic Serum. *Cancer Res.*, **27**, 1982.

STILLMAN, A. & ZAMCHEK, N. (1970) Recent Advances in Immunologic Diagnosis of Digestive Tract Cancer. *Am. J. dig. Dis.*, **15**, 1003.

STONEHILL, E. H. & BENDICH, A. (1970) Retrogenetic Expression: the Reappearance of Embryonal Antigens in Cancer Cells. *Nature, Lond.*, **228**, 370.

TAKAHASHI, A., YASHI, A., ANZAI, T. & WADA, T. (1967) Presence of a Unique Serum Protein in Sera Obtained from Patients with Neoplastic Diseases and in Embryonic and Neonatal Sera. *Clin. Chim. Acta*, **17**, 5.

TATARINOV, Y. S. (1965) The Content of an Embryospecific α-globulin in the Serum of Human Embryos, of New-born Babies and of Adults in Cases of Primary Carcinoma of the Liver. *Vopr. med. Khim.*, **11**, 20.

TEE, D. E. H., WANG, M. & WATKINS, J. (1965) Antigenic Properties of Human Tumours; "Tumour-specific" Antigens. *Eur. J. Cancer*, **1**, 315.

TEILUM, G. (1971) Special Tumors of Ovary and Testis. Copenhagen: Munskgaard. p. 33.

TERENT'EV, A. A. (1969) Comparative Study of Fetuin and Embryo-specific α-globulin in Bovine Fetal Serum. *Byull. eksp. Biol. Med.*, **68**, 740.

THOMSON, D. M. P., KRUPEY, J., FREEDMAN, S. O. & GOLD, P. (1969) The Radioimmunoassay of Circulating Carcinoembryonic Antigens of the Human Digestive System. *Proc. natn. Acad. Sci., U.S.A.*, **64**, 161.

TROUILLAS, P. (1971) Carcinofetal Antigen in Glial Tumours. *Lancet*, ii, 552.

TURNER, M. D., KLEINMAN, M. S. & HARWELL, L. (1970) Studies of Colonic Carcinoma Antigens. *Gut*, **11**, 1064.

URIEL, J., DE NECHAUD, B. & DUPIERS, M. (1972) Oestrogen-binding Properties of Rat Horse and Human Fetospecific Proteins by Immunoautoradiographic Methods. *Biochem. Biophys. Res. Comm.*, **46**, 1175.

WADA, T., ANZAI, T., YACHI, A., TAKAHASHI, A. & SAKAMOTO, S. (1971) Incidence of Three Different Fetal Proteins in Sera of Patients with Primary Hepatoma. In *Protides of the Biological Fluids.* Ed. H. Peeters. Oxford: Pergamon Press. p. 221.

WARNOCK, M. & REESMAN, R. (1969) Variant Alkaline Phosphatase in Human Hepatocellular Cancers. *Clin. Chim. Acta*, **24**, 5.

WEINTRAUB, B. D. & ROSEN, S. W. (1971) Ectopic Production of Human Chorionic Somatomammotrophin by Non-trophoblastic Cancers. *J. clin. Endocr.*, **32**, 94.

WISE, R. & OLIVER, I. (1966) Sites of Synthesis of Plasma Proteins in the Foetal Rat. *Biochem. J.*, **100**, 330.

WOOD, W. C. & MORTON, D. L. (1971) Host Immune Response to a Common Cell-surface Antigen in Human Sarcomas. *New Engl. J. Med.*, **284**, 569.

YASHI, A., MATSUMURA, Y., CARPENTER, C. M. & HYDE, L. (1968) Immunochemical Studies on Human Lung Cancer Antigens Soluble in 50% Saturated Ammonium Sulfate. *J. natn. Cancer Inst.*, **40**, 663.

ZAMCHEK, N., MOORE, T. L., DHAR, P. & KUPCHIK, H. (1972) Immunological Diagnosis and Prognosis of Human Digestive Tract Cancer: Carcinoembryonic Antigen. *New Engl. J. Med.*, **286**, 83.